# Two-photon time-lapse microscopy of BODIPY-cholesterol reveals anomalous sterol diffusion in chinese hamster ovary cells

**DOI:** 10.1186/2046-1682-5-20

**Published:** 2012-10-18

**Authors:** Frederik W Lund, Michael A Lomholt, Lukasz M Solanko, Robert Bittman, Daniel Wüstner

**Affiliations:** 1 Department of Biochemistry and Molecular Biology, University of Southern Denmark, Campusvej 55, Odense M, DK-5230, Denmark; 2 MEMPHYS Center for Biomembrane Physics, Department of Physics, Chemistry and Pharmacy, University of Southern Denmark, Odense M, DK-5230, Denmark; 3 Department of Chemistry and Biochemistry, Queens College, The City University of New York, Flushing, NY, 11367, USA

**Keywords:** Cholesterol, Transport, Fluorescence microscopy, Endocytosis, Vesicle, Tracking, Cytoskeleton dynamics

## Abstract

**Background:**

Cholesterol is an important membrane component, but our knowledge about its transport in cells is sparse. Previous imaging studies using dehydroergosterol (DHE), an intrinsically fluorescent sterol from yeast, have established that vesicular and non-vesicular transport modes contribute to sterol trafficking from the plasma membrane. Significant photobleaching, however, limits the possibilities for in-depth analysis of sterol dynamics using DHE. Co-trafficking studies with DHE and the recently introduced fluorescent cholesterol analog BODIPY-cholesterol (BChol) suggested that the latter probe has utility for prolonged live-cell imaging of sterol transport.

**Results:**

We found that BChol is very photostable under two-photon (2P)-excitation allowing the acquisition of several hundred frames without significant photobleaching. Therefore, long-term tracking and diffusion measurements are possible. Two-photon temporal image correlation spectroscopy (2P-TICS) provided evidence for spatially heterogeneous diffusion constants of BChol varying over two orders of magnitude from the cell interior towards the plasma membrane, where D ~ 1.3 μm^2^/s. Number and brightness (N&B) analysis together with stochastic simulations suggest that transient partitioning of BChol into convoluted membranes slows local sterol diffusion. We observed sterol endocytosis as well as fusion and fission of sterol-containing endocytic vesicles. The mobility of endocytic vesicles, as studied by particle tracking, is well described by a model for anomalous subdiffusion on short time scales with an anomalous exponent α ~ 0.63 and an anomalous diffusion constant of D_α_ = 1.95 x 10^-3^ μm^2^/s^α^. On a longer time scale (t > ~5 s), a transition to superdiffusion consistent with slow directed transport with an average velocity of v ~ 6 x 10^-3^ μm/s was observed. We present an analytical model that bridges the two regimes and fit this model to vesicle trajectories from control cells and cells with disrupted microtubule or actin filaments. Both treatments reduced the anomalous diffusion constant and the velocity by ~40-50%.

**Conclusions:**

The mobility of sterol-containing vesicles on the short time scale could reflect dynamic rearrangements of the cytoskeleton, while directed transport of sterol vesicles occurs likely along both, microtubules and actin filaments. Spatially varying anomalous diffusion could contribute to fine-tuning and local regulation of intracellular sterol transport.

## Background

Intracellular organelles contain very different amounts of cholesterol, but our knowledge about how these differences are established and maintained during continuous inter-compartment membrane traffic is very limited [1,2]. The dynamics of these processes can, in principle, be adequately addressed only by imaging-based approaches. This, however, is limited by the poor behavior of most fluorescent cholesterol analogs (reviewed in [3-6]). Ultraviolet (UV)-sensitive wide field (UV-WF) and multiphoton microscopy of the intrinsically fluorescent sterol dehydroergosterol (DHE) as a close analog of cholesterol and ergosterol has provided new insight into cellular sterol trafficking [7]. By this approach, it has been demonstrated that vesicular, ATP-dependent, and non-vesicular, ATP-independent, transport contribute to targeting of DHE from the plasma membrane to the endocytic recycling compartment (ERC) in various mammalian cells, although the relative contribution of each uptake mode differs between cell types [8,9]. Dynamics of intracellular vesicles containing DHE has been assessed by particle tracking of time-lapse sequences recorded on an UV-WF set-up. Calculation of the mean square displacement (MSD) from the trajectories as well as temporal image correlation spectroscopy (TICS) of images acquired at a multiphoton microscope indicated that diffusion of sterol vesicles in the cytoplasm is hindered, probably by cytoskeletal structures [7,10]. The poor fluorescence properties of DHE (UV emission, rapid bleaching, and low quantum yield), however, limited the availability of sufficient image data sets and thereby precluded an in-depth quantitative analysis of vesicular sterol transport.

A promising new cholesterol probe for some applications is BODIPY-tagged cholesterol (BChol), in which the borondipyrromethene fluorophore is situated in cholesterol’s aliphatic side chain [11,12]. We recently compared membrane partitioning and intracellular transport of DHE with that of BChol [13]. We found that DHE has a higher affinity for the liquid-ordered (lo) phase than BChol, but BChol still preferred this phase over the liquid-disordered (ld) phase (see Additional file 1: Figure S1 for the structure of both fluorescent sterols compared to cholesterol). We also showed that both sterols are targeted to the ERC, a major cellular sterol pool, with identical kinetics [13]. Uptake of both sterols from the cell surface was strongly reduced in baby hamster kidney (BHK) cells overexpressing a dominant-negative clathrin heavy chain, and co-internalization of BChol with fluorescent transferrin (Tf), a marker for this uptake pathway, could be demonstrated [13]. Both observations suggest that a large portion of plasma membrane sterol is internalized by clathrin-dependent endocytosis in these cells. BChol has also been used to study sterol trafficking in sphingolipid storage diseases [11] and to analyze lateral and rotational sterol diffusion in model membranes [14].

Two-photon (2P) excitation microscopy offers several advantages over the one-photon fluorescence microscopy approaches used so far to visualize BChol in living cells. The intrinsic sectioning capability of this technique combined with negligible bleaching propensity outside the focal region and deeper specimen penetration due to the long-wavelength-excitation enabled us to perform long-term tracking studies of vesicles containing BChol in live Chinese hamster ovarian (CHO) cells. In addition, we used raster image correlation spectroscopy (RICS) and TICS to map the diffusional mobility of BChol over entire cells. Single particle tracking applied to sterol vesicles was combined with mathematical modeling of the trajectories to decipher the diffusion modes under various conditions. We found evidence for anomalous subdiffusion of vesicles containing BChol on short time scales (t < ~5 s) with a transition to superdiffusion on longer time scales. We demonstrate that the superdiffusive mode is caused by directed transport along both microtubules and actin filaments. Confined diffusion of sterol vesicles might increase the likelihood for local non-vesicular sterol exchange by collision of the vesicles with surrounding organelle membranes.

## Results and discussion

### Comparison of diffusivity and availability of BChol in various cellular regions

**Figure 1:**
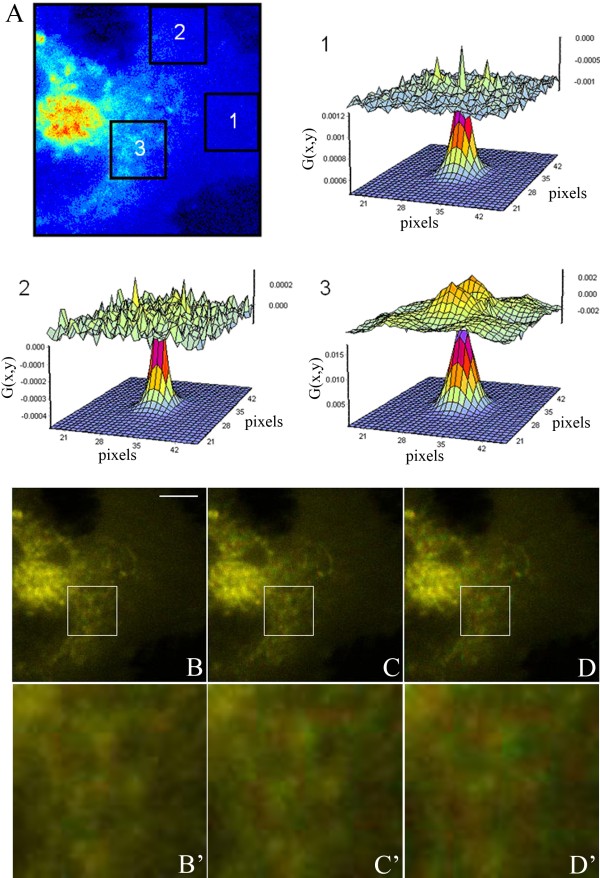
**Analysis of sterol mobility by raster image correlation spectroscopy (RICS).** CHO cells were pulse-labeled with BChol followed by a chase to obtain the steady-state distribution. The cells were placed on a temperature-controlled stage of a home-built 2P-microscope maintained at 35 ± 1°C, and images were acquired without pause at a frame rate of 0.52 s. Three areas of a cell incubated with BChol were analyzed with raster image correlation spectroscopy (RICS), panel **A**. The areas were selected to include the membrane (box 1), the cytoplasm with no apparent BChol vesicles (box 2), and an area close the ERC (box3). Inlet 1–3 show fits of the Brownian diffusion model to the RICS autocorrelation function in these areas and the residuals of the fit. The analysis showed that diffusion near the ERC was a factor of two slower than in the membrane. Nevertheless, the molecules in the central part of the cell were still mobile. This is illustrated in panels **B**-**D** (scale bar = 5 μm), which show the correlation between the cell at time t = 0 (colored red) with images at time t = 0.52, 5.2 and 10.4 s, respectively (colored green). The intracellular dynamics is especially apparent in panel **B’**-**D’** showing the dynamics in the ROI in panels **B**-**D**.

**Figure 2:**
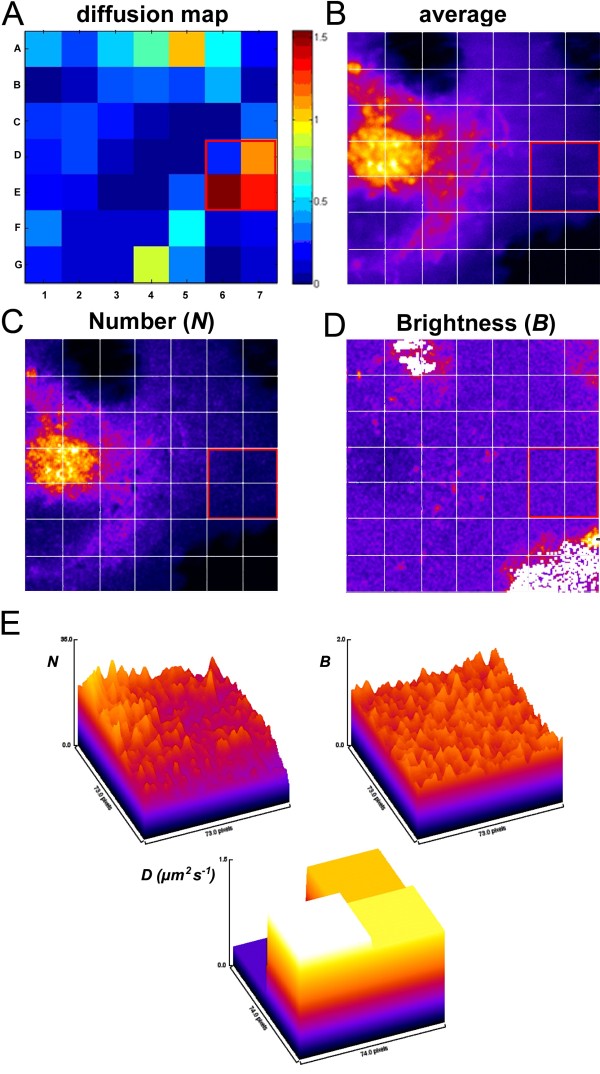
**Analysis of sterol mobility by temporal image correlation spectroscopy (TICS).** Cells were labeled and imaged as described in the legend to Figure 1. The intracellular transport of BChol was examined by temporal image correlation spectroscopy (TICS) and number and brightness analysis (**N**&**B**). Panel **A** shows a map of diffusion constants (**D**) in the cell ranging from ~1.5 – 0 μm^2^/s. Panels B-D show the average fluorescence intensity, the apparent number of molecules (**N**) and the apparent brightness (**B**), respectively. From a comparison of the average intensity and the apparent number of molecules we infer that the majority of BChol is found in the ERC. Although, the brightness map is relatively uniform (notice that the white areas are outside the cell) we still see signs of aggregation. This is most likely due to BChol binding transiently to intracellular structures (see text for discussion). Finally, Panel E shows the relationship between N, B and the diffusion constant of BChol. By comparison of N and D, it can be seen that the diffusion constant is inversely dependent on the number of molecules. As the brightness is constant over the ROI, we conclude that the decrease in diffusion constant is caused by molecular crowding rather than by aggregation of BChol.

Two-photon microscopy is, combined with fluorescence fluctuation techniques, ideally suited to determine the distribution of BChol with high spatiotemporal accuracy. In fluorescence fluctuation experiments the information is extracted from the small stochastic deviations of intensity, for example due to molecular diffusion or conformational changes, from the equilibrium intensity (mean value) in the focal volume. In 2P excitation one finds an intrinsic sectioning capability which stems from the non-linear excitation likelihood, i.e., that the photon densities required for 2P excitation are significant only in the focal spot. In confocal one-photon excitation microscopy, a pinhole in front of the detector rejects out-of-focus emission light, thereby greatly reducing the depth of field in the emission path. In contrast, excitation of the fluorophores takes place over a much larger distance along the optical axis than in 2P excitation microscopy, which increases the likelihood for photobleaching. We found that especially the photobleaching of sterol probes along the optical axis and the relatively lower sensitivity of our analog photomultiplier tubes make fluctuation experiments of BChol in one-photon excitation less reliable than in 2P excitation microscopy (not shown). Intensity fluctuations of the sterol caused by intracellular diffusion can be quantified by TICS and RICS in various cellular regions [15]. RICS uses the information contained within a single frame and correlates the rapidly scanned laser beam with intensity fluctuations during one scan over the sample. Thus, RICS has the advantage of being able to measure diffusion times in the range 10^-5^ s to 10^-0^ s [16-18]. First, we applied RICS to determine the diffusion coefficient in three 38.44 μm^2^ areas of the cell shown in Figure 1. The areas were selected to include 1) the outer rim of the cell likely being dominated by lateral diffusion of the sterol in the plasma membrane, 2) a region with intermediate thickness missing discernible vesicular structures and 3) the tubovesicular network emanating from the perinuclear ERC pool (see boxes numbered 1 to 3 in Figure 1A). This analysis yielded diffusion coefficients D_1_ = 1.267 μm^2^/s, D_2_ = 0.923 μm^2^/s, and D_3_ = 0.095 μm^2^/s, for boxes 1, 2 and 3, respectively. From the RICS correlation functions calculated for each box, and plotted with the residuals of the fit of the diffusion model (Figure 1A), one can infer that the fit quality is better for box 1 and box 2 compared to box 3. The lower fit quality in box 3 is likely a consequence of the dynamic properties of the sterol-containing tubules deviating from purely Brownian motion. Although diffusion in the central area of the cell is significantly slower than in the periphery of the cell, the molecules are not immobile. This is illustrated in Figure 1B-D which show a color overlay of the first frame (t = 0, in red) and the frames recorded after 0.52, 5.2, and 10.4 s (in the green channel of an RGB merge in ImageJ), respectively. In that representation, coincidence of vesicles will appear orange, while for increasing displacement of vesicles over time, separate red and green spots will appear. Figure 1A’-C’ show a zoom on the central area of the cell where increasing displacement over time is apparent. To assess the heterogeneity of diffusion in a more systematic way, we performed a 2P-TICS analysis on the same image sequence mapped over the entire cell. In TICS, the time autocorrelation function is determined as a measure of the average spatial correlation between images in a time series. The images can be subdivided into local regions of interest (ROIs) over which the averaging is performed. In this way, spatially heterogeneous probe dynamics can be resolved. The local diffusion coefficient of BChol determined for 32 x 32 pixel ROIs (10.24 μm^2^) using 2P-TICS varied from 0.05 – 0.5 μm^2^/s adjacent to the ERC toward 1.5 μm^2^/s in the periphery of the cell (Figure 2A). Thus, RICS and TICS performed on the same data set provide comparable diffusion constants for BChol. To gain insight into the molecular mechanisms underlying spatially varying sterol diffusion, we performed a number and brightness (*N*&*B*) analyses. *N*&*B* is a statistical method for investigating intensity fluctuations in fluorescence microscopy image sequences based on the experimentally determined distribution of emitted photons [15,19,20]. While photon arrival times are Poisson distributed, intensity fluctuations due to monomers or probe aggregates moving through the laser focus result in broadening of the histogram [19,20]. The first and second moment of a Poisson distribution are equal (i.e. mean is equal to variance), such that the apparent brightness defined as the ratio of the 2^nd^ and 1^st^ moment equals one. This situation corresponds to immobile excited fluorophores emitting photons by a stochastic Poisson process. If all fluorescent probes exist as monomers and are mobile, their apparent brightness becomes slightly larger due to the additional fluctuations in the photon counts, broadening the photon count distribution (i.e., increasing the variance). The brightness distribution over the analyzed image region will still consist of a single population. If additional aggregates of the fluorescent molecules form, a second population with higher values of the apparent brightness will be observed. Thus, the measured intensity fluctuations of BChol relate to the brightness and number of the fluorescent sterols in the focal volume (see Eq. 6 and 7 in Materials and Methods). N&B analysis has also been applied to determine spatial variations in concentration and degree of aggregation of fluorescent proteins in living cells [19,21-23]. Recently, we used N&B analysis to provide evidence against lateral domains of DHE in the plasma membrane of HepG2 cells [7]. Here, we apply the method to intensity fluctuations of BChol (Figure 2). The average fluorescence intensity (‘average’), apparent number of molecules of BChol (‘Number (N)’), and their apparent brightness (‘Brightness (B)’) are shown in Figure 2B, C, and D, respectively. The high coincidence of the average and *N*-map indicates that intracellular regions with high BChol fluorescence contain proportionally many molecules. Most BChol molecules are located in the ERC and in its associated vesicles surrounding the ERC [13]. The B-map provides information about potential clustering or mobile assemblies of a fluorophore, since probe aggregates would have proportionally higher brightness for a given fluorescence fluctuation [19,20]. The B-map of BChol is relatively uniform, especially in the plasma membrane, which indicates the absence of sterol domains. We obtained the same result in a separate set of experiments carried out with HeLa cells (Additional file 1: Figure S2). In both cell types, CHO and HeLa cells, only one population of B-values was measured in the plasma membrane with a mean value of B ~ 1.01-1.05 (not shown). This is characteristic for mobile monomers, as previously demonstrated for monomeric enhanced green fluorescent protein (eGFP) expressed in CHO cells [19]. For BChol, bright fluorescent spots were only observed in the area surrounding the perinuclear recycling endosome (Figure. 2D). These spots are likely due to slowly moving BChol enriched vesicles (compare Figure 1B’-D’). Immobile vesicles would not give an increase in the *B*-maps, according to theory (see above and Supplemental Information). We also ruled out spatially varying photobleaching of BChol as cause for the observed brightness differences, since BChol showed no photobleaching under 2P excitation during our measurements (Additional file 1: Figure S3). Since BChol exists as fluorescent monomers and as part of vesicles in the cytoplasm, we performed a stochastic simulation of Brownian diffusion with transient binding to slowly moving vesicle-like structures (see Additional file 1: Figure S4). Subsequent analysis by TICS as well as N&B analysis revealed a strong similarity between the simulated and experimental data (compare Figure 2 and Additional file 1: Figure S5). To test other scenarios, we performed additional simulations (Additional file 1: Figure S6-S8). First, sterol containing vesicles and sterol monomers were considered with an equilibrium distribution based on the first simulation but without any exchange between both sterol pools. This resulted in homogeneous N- and B-maps with no sign of clustering of sterol monomers around the slowly moving sterol vesicles (Additional file 1: Figure S6). Second, we kept the binding/dissociation rate constants fixed but increased the diffusion constant of the sterol monomers in the cytoplasm (compare Additional file 1: Figures S5 and S7). At non-physiologically high diffusion constants of monomers of 300 μm^2^/s, the simulated sterol monomers could escape the binders/vesicles and therefore no sterol accumulation in vesicles was found. Finally, increasing the binding strength of sterol monomers to vesicles by a factor of 10 compared to the first simulation resulted in large sterol clustering and a structured B-map (compare Additional file 1: Figures S5 and S8). Together, the simulations provide one explanation being congruent with the experimental data, namely that the spatially heterogeneous diffusion and concentration of BChol is caused by transient sterol binding to immobile or slowly diffusing macromolecular structures in the cytoplasm. Further work in our laboratory is set out to directly test this hypothesis and to validate the model. For a detailed description of the model/simulation as well as the MatLab code for the simulation program, see the Supplementary Information. The relationship between the N-map, B-map and diffusion coefficient, *D*, is shown for the experimental data of BChol in a 62x62 pixel ROI (38.44 μm^2^) composed of area D6, D7, E6, and E7 (Figure 2A) in the periphery of the cell where molecules are scarce. In this area, the measured diffusion coefficients show an inverse relationship to the *N*-map, the apparent number of particles. This can be clearly seen in the 3D plots of Figure 2E, where only the region with a higher number of molecules in the left corner, close to the origin (area D6, compare with Figure 2A) has a low diffusion constant. Since the B-map is homogeneous for all four regions, the slowed diffusion in area D6 means that there are proportionally more BChol molecules, likely due to a contribution from plasma membrane and cytoplasm. The other three quadrants in Figure 2E (i.e., area D7, E6, and E7; compare with Figure 2A) resemble mostly the plasma membrane, which has the highest sterol diffusion coefficients. Since diffusion is slower in areas with increased N-values (Figure 2E), we cannot rule out some contribution from molecular crowding in areas of the cell where the concentration of molecules is high.

### Dynamics of vesicles containing BChol reveal anomalous diffusion characteristics

**Figure 3:**
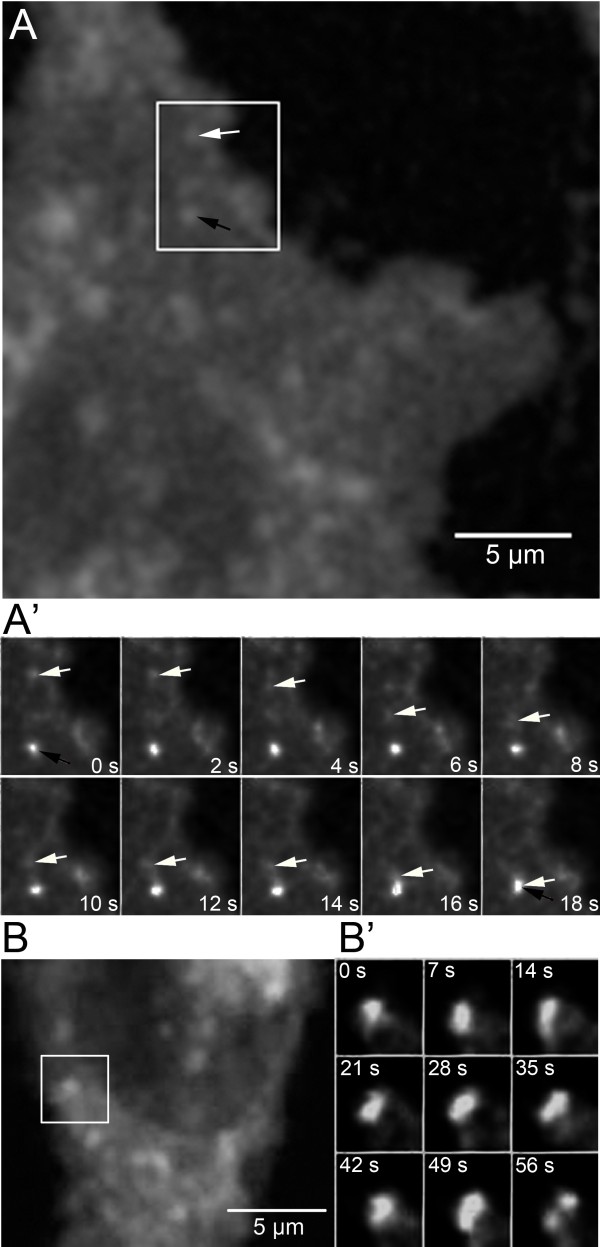
**Two-photon time-lapse imaging of fusion and fission of vesicles containing BChol.** CHO cells were pulse-labeled with BChol followed by a chase to obtain the steady-state distribution. The cells were placed on a temperature-controlled stage of a home-built 2P-microscope maintained at 35 ± 1°C, and images were acquired without pause at a frame rate of 1.05 s. We observed vesicle fusion (**A’**): one vesicle (white arrow) moved with a directed motion toward another vesicle (black arrow) over a period of 18 s. Further observation of the fused vesicle over 100 s did not show subsequent fission of the vesicle. We also observed vesicle fission (**B**, **B’**): over a period of ~1 min the vesicle was heavily stretched and deformed before it finally separated into two vesicles with lower fluorescence intensity.

To determine the mobility of sterol-containing vesicles, we employed multiple particle tracking on image sequences obtained by 2P microscopy. CHO cells were pulse-labeled with BChol followed by a chase to obtain the steady-state distribution. It has been shown by others that esterification of BChol using a cyclodextrin-based labeling protocol and chase times up to 2 h in various cell types including CHO cells is negligible (i.e., below 1% of total cell-associated BChol) [11]. The cells were placed on a temperature-controlled stage of a home-built 2P-microscope maintained at 35 ± 1°C, and images were acquired without pause at a frame rate of 1.05 s. The high spatiotemporal resolution of BChol in 2P-time-lapse microscopy allowed us to observe fusion of two sterol-containing vesicles adjacent to the plasma membrane (Figure 3A). Another example from the same data set can be found in [24]. In addition to fusion, we also observed splitting of one sterol vesicle into two (Figure 3B) and shuttling of a small carrier vesicle between two larger vesicular structures (Additional file 1: Figure S9), all containing BChol. Together, we occasionally observed fusion and fission of sterol-containing endocytic vesicles as well as shuttling of small vesicles containing BChol between larger vesicles (together about 10 such events were found in more than 300 observed/tracked vesicles). The low incidence of such events, however, makes a major contribution to intracellular sterol sorting unlikely.

**Figure 4:**
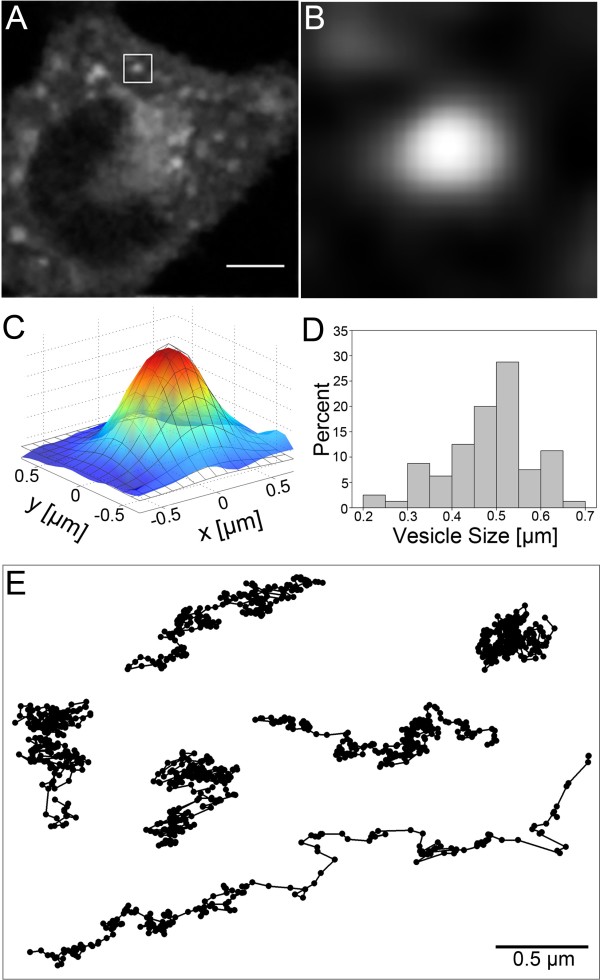
**Estimation of vesicle size and example trajectories of vesicles containing BChol.** After cells were labeled and chased with BChol, as described in the legend to Figure 3, vesicles were found throughout the cytoplasm with the majority of vesicles in the perinuclear region (**A**). The average vesicle size was determined by fitting a 2D Gaussian function to images of the vesicles. Panel **B** shows a vesicle from the ROI in panel **A** (image size 2.4 x 2.4 μm) while panel **C** shows a fit of the 2D Gaussian to the vesicle. The vesicle sizes had a distribution with diameters ranging from 0.2 – 0.7 μm with an average size of 0.45 ± 0.1 μm (panel **D**). To monitor their mobility, vesicles were tracked and their trajectories analyzed. Panel **E** shows examples of vesicle trajectories. Bar, 5 μm.

By visual inspection as well as detailed tracking analysis (see below), we found that the majority of vesicles containing BChol were moving within an area of 1 × 1 μm without major directed displacement. Diffusion in a limited cellular domain could indicate anomalous subdiffusion due to some barriers. Tracking of lipid granules as well as inert tracer particles in yeast, mammalian cells, and bacteria has shown that particles of similar size to endocytic vesicles move by anomalous subdiffusion [25]. This is partly caused by the ability of the cytoskeleton to capture the particles, and partly by molecular crowding by other macromolecules [26-29]. Experiments on semi-dilute solutions of actin revealed an entangled network of semi-flexible polymers with an average mesh diameter of the order 100 nm to 1 μm, in which colloidal tracer particles moved by anomalous subdiffusion [30]. This anomalous diffusion was caused by local jumps between micro-environments driven by elastic forces of the actin network [30]. Particles smaller than the average mesh size moved by normal diffusion but with reduced diffusion constant due to the local viscosity and crowding effects. Based on these observations, we wanted to estimate how likely it is that vesicular sterol transport is hindered by the cytoskeleton meshwork resulting eventually in anomalous diffusion. We fitted a two-dimensional Gaussian function to images of the vesicles and thereby determined the size of BChol vesicles in the cytosol (Figure 4A-D). We found values for the full width at half maximum (FWHM) ranging from 0.2 – 0.7 μm with an average value of 0.45 ± 0.1 μm (Figure 4D). In independent experiments, we estimated the FWHM for sub-resolution quantum dots with our multiphoton set up to be ~0.28 μm as the estimated width of the point spread function [7]. Accordingly, a significant fraction of vesicles containing BChol are larger than the resolution limit of the microscope, and their size can, therefore, be measured exactly. Previous size measurements from microscope images gave comparable values for GLUT4 storage vesicles (FWHM = 0.36 μm) in 3 T3-L1 cells [31] and secretory granules in chromaffin cells (FWHM = 0.38 μm) [32]. With an average diameter of 0.45 μm, the size of the BChol vesicles is on the order of the mesh size of actin filaments (see above). Thus, it is likely that diffusion of sterol vesicles is hindered by the cytoskeleton, causing deviation from pure Brownian motion.

To examine this notion, we analyzed the mobility of sterol-containing vesicles by multiple particle tracking and subsequent analysis of their average MSD. Figure 4E shows examples of vesicle trajectories from vesicles in control cells at 37°C. Various types of vesicle trajectory shapes were observed ranging from almost isotropic trajectories, i.e., without a preferred direction of displacement, to stretched or elongated trajectories, the latter characteristic for directed motion (compare the upper right corner and lower trajectory in Figure 4E). Directed motion results in correlations between the displacements in x- and y-direction which manifests in non-zero off-diagonal elements in the diffusion tensor [33]. For pure Brownian motion, the MSD of the trajectory is directly proportional to time and subsequent steps are not correlated. Anomalous diffusion, on the other hand, is described by an anomalous exponent, α, where the MSD is proportional to *t*^α^. For 0 < α < 1 subdiffusion, occurs with more restricted mobility as α decreases. Anomalous subdiffusion of tracer particles and lipid granules as a consequence of particle confinement has been described by fractional Brownian motion (FBM) [34,35]. The main difference between Brownian motion and FBM is that in the latter the steps are correlated, while in the former mode of motion, they are not. For example, in restricted or confined diffusion, a step is more likely to be in the opposite direction of the former step. This (anti)-correlation is described for FBM by a power law where the value of α determines the type of motion (i.e., subdiffusion, α < 1 or superdiffusion with α > 1). Since we also observed vesicles with fast directional displacement (see the lower trajectory in Figure 4E for an example), we extended the subdiffusive FBM model to include an additional ballistic term describing the transition from subdiffusion to directed transport:

(1) $$\begin{array}{rl} {〈{\vec{x}}^{2}\left(t\right)〉}_{\vec{f_{0}}} & ={〈{\vec{x}}^{2}\left(t\right)〉}_{0}+{〈\vec{x}\left(t\right)〉}_{\vec{f_{0}}}^{2}\\ & =4D_{\alpha }t^{\alpha }+v^{2}t^{2}\text{,} \end{array}$$

**Figure 5:**
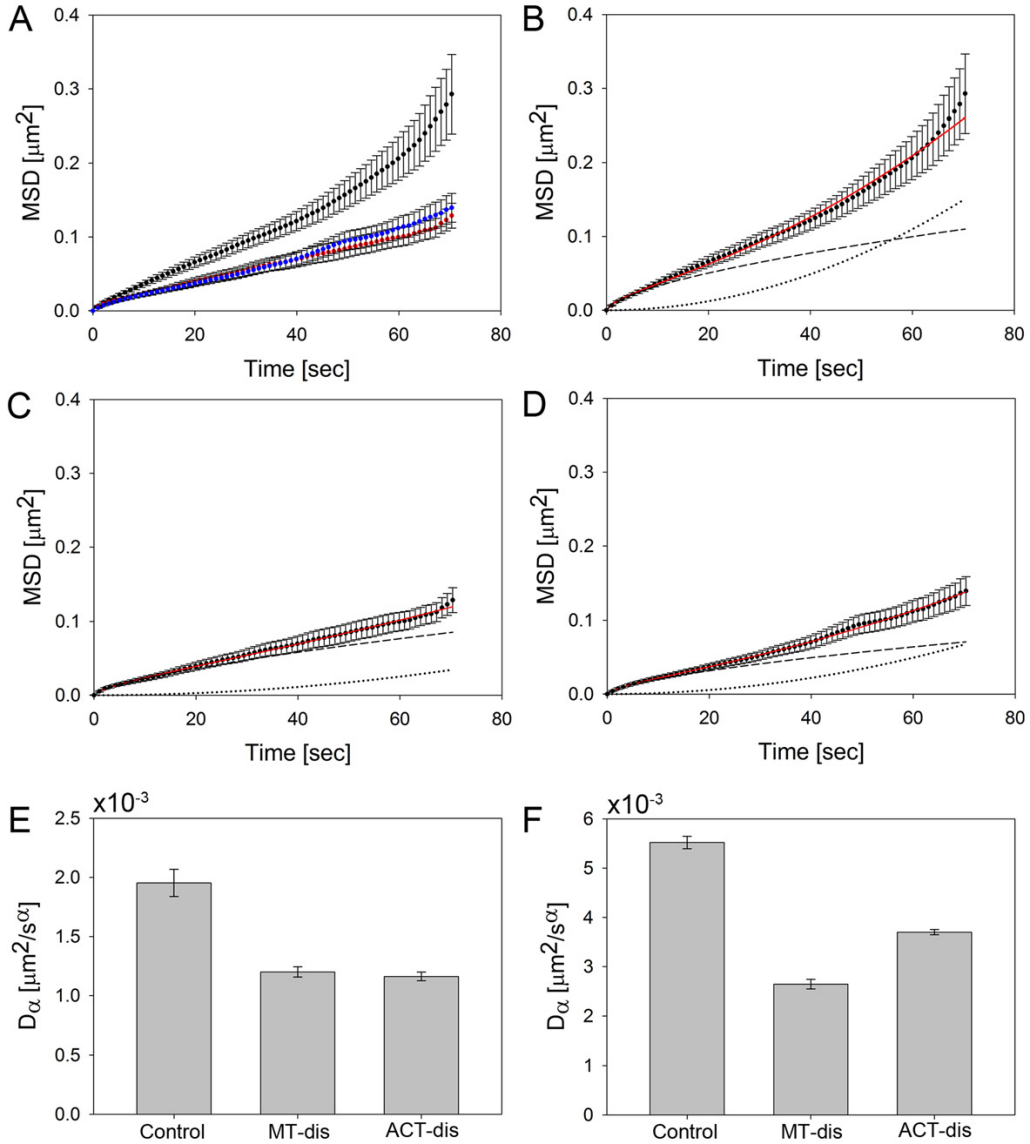
**Quantitative analysis of all trajectories of vesicles containing of BChol.** CHO cells pre-incubated in the absence or presence of Cytochalasin D, to disrupt the actin cytoskeleton, or nocodazole, to disrupt microtubule, were pulse-labeled with BChol followed by a chase in the presence or absence of the drugs to obtain the steady-state distribution. The cells were imaged as described in the legend to Figure 3. Vesicles were tracked as described in Materials and Methods and their mobility was analyzed by the average mean square displacement (MSD) averaged over all vesicle trajectories per condition. Disruption of microtubule (panel **A**, red) or actin filaments (panel **A**, blue) significantly reduced vesicle mobility compared to the mobility of vesicles in control cells (panel **A**, black). Vesicle mobility was analyzed by a model describing the transition from anomalous subdiffusion to directed motion (Eq. (1)). Panels **B**, **C**, and **D** show the regression of this model (red line) to the MSD for vesicles in control cells (panel **B**) and in cells with disrupted microtubule (panel **C**) or disrupted actin filaments (panel **D**). Also shown are the contribution from anomalous subdiffusion (dashed lines) and superdiffusion consistent with directed motion (dotted lines). Disruption of both microtubule and actin filaments reduced the anomalous diffusion constant and the velocity of the directed motion. Notice that as the anomalous diffusion constant, D_α_, depends on the anomalous exponent the comparison in panel E is only valid as we find α to be constant. Data is given as mean ± SD being calculated as described in Eq. 5 in Materials and Methods.

Here, *D*_*α*_ is the anomalous diffusion constant, α is the anomalous exponent, defined above, *v* is the velocity of the directed transport and the subscript $\vec{f_{0}}$ indicates the presence of a pulling force (see Materials and Methods, Eqs. 3 and 4 for details). Fitting Eq. 1 to the average MSD of 210 vesicle trajectories from 17 control cells monitored at 37°C yielded an anomalous diffusion constant, *D*_*α*_ = 1.95 × 10^− 3^*μm*^2^/*s*^*α*^, an anomalous exponent α = 0.62 and a velocity *v* = 5.52 x 10^-3^ μm/s (see Materials and Methods for a description of the fitting procedure). Figure 5B shows the MSD for vesicles in control cells (black dots) and the curve for the fit of Eq. 1 with error bars showing the standard error. The dashed and dotted lines show the contributions of subdiffusion and directed transport, respectively (compare Eq. 1 with Eqs. 3 and 4). On short time scales, the MSD plot is curved downward due to the anomalous subdiffusion (dashed line, corresponding to the first term on the right-hand side (RHS) of Eq. 1). On longer time scales, superdiffusion consistent with active transport results in an upward curved MSD plot given by the dotted line and the second term on the RHS of Eq. 1 (see above and Figure 5B-D).

Anomalous subdiffusion is frequently observed for various cargo molecules in cells and is generally ascribed to the dynamics of the cytoskeleton with typical values of α ranging from α ~ 0.65-0.75, [26,36-38]. Thus, the anomalous exponent we find for sterol-containing vesicles is slightly smaller than that reported for other vesicle cargo, but since the anomalous exponent was found to vary between different cell types, the value we find here is still within the expected range. The average transport velocity of endosomes and other vesicles along microtubules has been estimated to range from 0.25 μm/s to 2 μm/s [39-43]. Thus, the average velocity we find here for BChol-containing vesicles in CHO cells (i.e., 5.52 x 10^-3^ μm/s) is roughly a hundred times slower, indicating that directed transport plays a minor role in vesicular sterol trafficking in CHO cells. In references [44,45], the velocity of vesicle transport was determined by selectively measuring the velocity of vesicles being actively transported. Here, on the other hand, we determine the average mobility of a large population of vesicles containing a small but significant subpopulation of vesicles being actively transported through the cytoplasm. Thus, the much slower average velocity we find here is likely due to a different experimental approach. Visual inspection of the data showed that few vesicles were almost entirely subject to directed transport, while the majority of vesicles containing BChol showed no directional transport over the observation time; see Figure 4E for examples of trajectories. Thus, it is likely that two differently mobile vesicle populations transport BChol in CHO cells. Analysis of the end-to-end distance of vesicle trajectories indicates that the population of vesicles with predominantly active transport is very small (Additional file 1: Figure S10). In fact, the majority of vesicle trajectories have an end-to-end distance of less than 1 μm, and out of the 210 vesicles tracked at 37°C in control cells, only eight vesicles had an end-to-end distance of more than 1 μm, up to ~3.2 μm; (see Additional file 1: Figure S10). We cannot rule out that these vesicles carry out special functions in intracellular sterol trafficking. Long-range active transport of endocytic vesicles primarily occurs along microtubules while actin filaments have been shown to support local short-distance vesicle movements [44,45]. Furthermore, as discussed above, the filaments of the cytoskeleton should impact or even determine the diffusion modality of sterol vesicles (normal versus anomalous diffusion). Therefore, we proceeded to uncover the effects of disrupting either the microtubule or the actin filaments. Microtubule disruption was induced by treating the cells for 1 h with 33 μM nocodazole while actin was disrupted by incubation with 20 μM cytochalasin D for 1 h [46]. Figure 5A shows the MSD after microtubule disruption (red, n = 132 vesicles from 15 cells) and actin disruption (blue, n = 111vesicles from 15 cells) compared to the MSD of vesicles in the control cells (black). It is evident that both treatments significantly reduced vesicle mobility. A fit of Eq. 1 to both MSDs showed that neither microtubule nor actin disruption altered the degree of anomaly significantly. In both cases, we found α = 0.65, while in control cells with an intact cytoskeleton the anomalous exponent was α = 0.62. Interestingly, the lowered MSD upon disruption of microtubules or actin filaments was caused by both a decrease in diffusion constant and by a decreased velocity. After microtubule disruption, the anomalous diffusion constant was reduced by 39% and the velocity by 52%. Similarly, the anomalous diffusion constant and the velocity were reduced by 40% and 33%, respectively, after disruption of the actin filaments; see Figure 5E, 5F. We infer that sterol-containing vesicles are transported along both microtubules and actin filaments although disruption of microtubules seems to have a slightly larger effect on active transport.

## Conclusions

**Figure 6:**
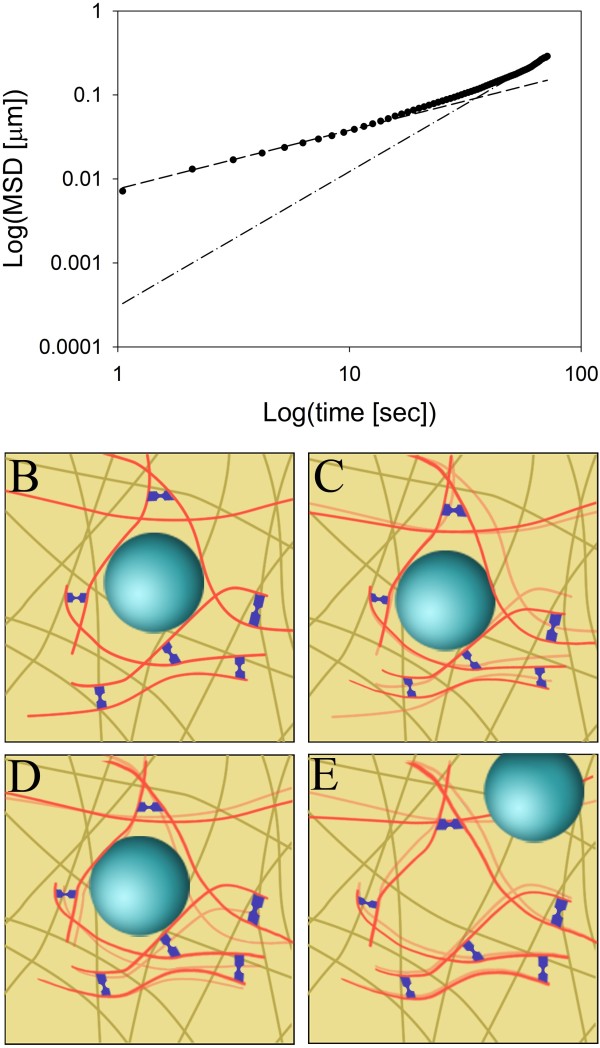
**Suggested model for diffusion of sterol-containing vesicles.** (**A**) Log-Log plot of the average MSD of vesicles tracked in control cells at 37°C. Initially, the MSD has a slope described by anomalous subdiffusion (dashed line) with an eventual transition to a slope described by superdiffusion consistent with active transport (dotted line). From this, we suggest the model presented in panels B-E. On short time scales the vesicles are caught in the meshwork formed by the cytoskeleton, which is composed of microtubule and actin filaments. This meshwork of filaments undergoes thermal fluctuations and is interconnected by motor proteins, which are constantly pulling on the filaments. Thus, the cytoskeletal network is constantly rearranging, causing the vesicles to appear to move by anomalous subdiffusion. On longer time scales directional motion caused by motor proteins moving along a filament dominates, thus causing a directed vesicle motion on longer time scales.

In summary, we propose the model shown in Figure 6 for the mobility of BChol-containing vesicles in CHO cells. On short time scales (t < ~5 s), the vesicles are caught in the cytoskeleton either in cages or directly attached to a given filament, resulting in anomalous subdiffusion of vesicles (Figure 6B-D).We used a frame rate of ~1 Hz for our tracking studies, which is comparable or even faster than many other vesicle tracking studies with fluorescent probes [39,47,48], even though some vesicle tracking studies use much higher frame rates up to 30 Hz [49]. In our previous work on tracking of sterol vesicles using DHE on a WF microscope, much lower frame rates of ~0.015-0.03 Hz were used to catch significant vesicle displacements in the presence of the unavoidable photobleaching of that sterol probe [3,10]. We found the time resolution of ~1 frame/s used here sufficient to accurately describe motion characteristics of BChol, and we would predict that finer temporal sampling only minimally affects estimation of the diffusion parameters given the low displacement distances we observe in the subdiffusive mode for (t ≤ 5 s). The observed subdiffusive motion of the vesicles is likely a consequence of dynamic rearrangements of the cytoskeleton sampled with a similar time resolution as we used here [50], which behave as an elastic polymer network, as recently suggested by Brangwynne et al. [51]. In CHO cells, we found an anomalous exponent, α ~ 0.63, which is slightly smaller than the anomalous exponent for lipid droplets in yeast [26]. Anomalous subdiffusion would also be possible in a static meshwork. Here, the vesicles could be caught in cages formed by the cytoskeleton, but they would still be able to move by confined diffusion. However, based on the results presented here this is not likely for two reasons. First, we find that the average vesicle size corresponds to the average distance between filaments in the cytoskeleton. Therefore, it is likely that in a static meshwork the vesicles would not have space for diffusion but could only move by active transport along filaments. Second, the anomalous exponent for vesicle motion closely resembles the anomalous exponent observed for the mobility of a dynamic cytoskeleton [36-38,52]. This further strengthens that anomalous subdiffusion of the vesicles on short time scales is likely due to rearrangements of the cytoskeleton. On longer time scales, the vesicles have a probability of being transported by one or several motor proteins which pull the vesicles across cytoskeleton barriers. Thus, we find a switch to superdiffusive motion along a given filament (illustrated in Figure 6E by a large displacement) as inferred from fitting Eq. 3 to the last third of the MSD (i.e. large t). This analysis gives an anomalous constant of 1.6 (see dotted-dashed line in Figure 6A after transforming data and fitting to a log-log plot). Interestingly, while most of the literature regarding transport of endocytosed vesicles has focused on transport along microtubules, we find that transport of sterol-containing vesicles is reduced by disruption of either microtubules or actin filaments. This suggests that actin filaments are linked to transport of sterol-containing vesicles, as suggested previously for DHE in vesicles containing the putative sterol transporter MLN64 [53]. However, if this is due to vesicles being transported along actin filaments or due to some restructuring of the cytoskeleton is yet to be determined.

### Significance of the results for regulation of intracellular cholesterol transport

It is well documented that different intracellular organelles contain very different amounts of cholesterol. However, much remains to be understood about how this spatiotemporal distribution is maintained. Using two-photon microscopy of the fluorescent cholesterol analog BChol, we found that diffusion of non-vesicular sterol in cells is very heterogeneous with diffusion constants ranging from 10^-2^ μm^2^/s close to the ERC to 1.3 μm^2^/s towards the plasma membrane. This non-vesicular sterol diffusion is likely interrupted by transient binding of BChol monomers to slowly moving vesicles (see Figures 1, 2 and Additional file 1: Figures S5 to S8). By particle tracking analysis we demonstrate that transport of vesicles containing BChol is governed by anomalous subdiffusion on a time scale less than ~5 s with a transition to superdiffusive motion consistent with directed transport on longer time scales. Both processes require an intact actin and microtubule network. The slow transport velocity in the superdiffusive mode indicates that active transport of vesicles containing sterols is not a major factor in vesicular sterol transport. Rather, most sterol vesicles move in small confined areas. This has likely two consequences. First, the predominantly subdiffusive motion of sterol vesicles in small confinement areas make inter-organelle vesicular sterol transport including vesicle budding, shuttling to a target organelle and fusion of the vesicle with the target organelle an inefficient transport mode. Budded vesicles will likely stay close to the donor membrane for prolonged time, as predicted for subddiffusive motion [54]. Second, anomalous subdiffusion of sterol vesicles could increase the time for local collisional sterol transfer to adjacent organelles. In fact, exchange of cholesterol monomers between donor and acceptor liposomes has been shown to be enhanced by frequent vesicle collisions [55]. We propose that confined diffusion of sterol vesicles in small subcellular domains increases the likelihood of local non-vesicular sterol exchange, either due to collisions of vesicles with adjacent membranous structures or by transport via sterol carrier proteins. This of course assumes that the sterol carrier proteins may pass through the barrier that confines the vesicle. The proposed mechanism could be an elegant way of coupling vesicular and non-vesicular sterol transport modes in mammalian cells.

## Methods

### Cell culture and labeling

Chinese Hamster Ovary (CHO) cells were grown in bicarbonate-buffered Ham’s F12 medium supplemented with 5% heat-inactivated fetal calf serum and antibiotics as previously described [8]. Fetal calf serum and cell culture medium were purchased from Gibco BRL (Life Technologies, Paisley, Scotland), while all other chemicals except BChol were from Sigma Chemical (St. Louis, MO). Two to three days prior to experiments, cells were seeded on microscope slide dishes. Lipid probes were stored in ethanol at a concentration of 5 mM under nitrogen at – 80°C until use. BChol was synthesized and loaded on methyl-β-cyclodextrin as described previously, affording a solution containing BChol/cyclodextrin complexes (BChol-CD) [13]. Cells were labeled with BChol-CD for 2 min at 37°C, washed with buffer medium containing 150 mM NaCl, 5 mM KCl, 1 mM CaCl_2_, 1 mM MgCl_2_, 5 mM glucose and 20 mM HEPES (pH 7.4) and chased for 25 min at 37°C prior to imaging. Microtubule disruption was induced by treating the cells for 1 h with 33 μM nocodazole while actin was disrupted by incubation with 20 μM cytochalasin D for 1 h [46].

### Two-photon excitation microscopy

Fluorescence time lapse measurements of BChol were performed using a custom-built setup constructed around an Olympus IX70 microscope. The objective used was a 60x water immersion objective with a NA of 1.2. The excitation light source was a femtosecond Ti:Sa laser (Broadband Mai Tai XF W25 with a 10 W Millennia pump laser, 80 MHz pulse-frequency, tunable excitation range 710–980 nm, Spectra Physics, Mountain View, CA), and the excitation wavelength used was 930 nm. To collect BChol’s emission, a 540 ± 25 nm filter was used (BrightLine HC). The light was detected by a photomultiplier tube (Hamamatsu H7422P-40) operated in the photon counting mode. The data were acquired using simFCS software developed by the Laboratory for Fluorescence Dynamics, University of California, Irvine.

### Image analysis

Image analysis was carried out using ImageJ (developed at the U.S. National Institutes of Health and available on the Internet at http://rsb.info.nih.gov/ij) or MatLab (MathWorks Inc., USA). For spatial registration of image stacks, “StackReg” developed by Dr. Thevenaz at the Biomedical Imaging group, EPFL, Lausanne, Switzerland was used [56]. Prior to multiple particle tracking, the images were processed to enhance the signal to noise ratio by the PureDenoise algorithm [57,58] for ImageJ and a 0.5 Gaussian filter applied to the image sequences. After removal of residual background, the image was processed with the spot enhancing Mexican hat filter implemented in the SpotTracker plugin for ImageJ by Daniel Sage [59].

### Multiple particle tracking

Tracking of vesicles was performed using the 3D tracking plugin for ImageJ by Sbalzarini and Koumoutsakos [60]. In case of translational motion of the cell, this was corrected for by image registration using the StackReg plugin for ImageJ.For each vesicle the x- and y-coordinates in each frame were saved for further analysis in MatLab v. 7.9.0 (R2009B) using self-programmed routines. The mean square displacement (MSD) was calculated as:

(2) $$\begin{array}{ll} MSD(\Delta t & =nh)=\frac{1}{N-n}\sum_{i=1}^{N-n}\\ & \left\{{\left[x\left(ih\right)-x\left(ih+nh\right)\right]}^{2}+\left[y\left(ih\right)-y\left(ih+nh\right)\right]\right\} \end{array}$$

where *N* is the number of frames, *h* is the time step between subsequent frames, and Δ*t* is the time lag corresponding to n frames.

### Model for combined subdiffusion and active transport

In our model for the vesicle motion we combine subdiffusion, which could be of the type fractional Brownian motion, with a constant velocity deterministic movement due to pulling by the motor proteins. The MSD for the subdiffusive motion is:

(3) $${〈\vec{x^{2}}\left(t\right)〉}_{0}=4D_{\alpha }t^{\alpha }\text{.}$$

The pulling of the motor proteins is assumed to shift the average position by:

(4) $${〈\vec{x}\left(t\right)〉}_{\vec{f_{0}}}=v\cdot t\text{,}$$

where the subscript $\vec{f_{0}}$ indicates the presence of the pulling force. We take the diffusion and the force as independent and, thus, the MSD in the presence of the force is simply the sum of Eq. (3) and the square of Eq. (4) giving Eq. (1) in the results and discussion section. Due to the way the MSD is calculated it is statistically more significant for small lag times. In fact, for a purely diffusive process, the standard deviation of the MSD is proportional to the MSD itself [33]. Thus, the MSD fit was weighted by 1/(*Stderr*)^2^, where *Stderr* is the standard error of the MSD see error bars in Figure 5. In MatLab this yields a fit with an associated 95% confidence interval corresponding to 1.96 standard deviations. Thus, the standard deviation, *Std*, of the fitted value is given by:

(5) $$Std=\frac{CI-\overset{―}{x}}{1.96}\text{,}$$

where *CI* is the upper 95% confidence interval and $\overset{―}{x}$ is the mean fitted value.

### Temporal image correlation spectroscopy (TICS)

TICS correlates an image series in time to determine the dynamics, number densities, and fraction of immobile fluorophores on the time scale of the measurement [18,61]. The normalized temporal autocorrelation function of an image series as a function of time lag τ, when ξ = η = 0 is given by [18,62]:

(6) $$r\left(0,0,\tau \right)=\frac{〈\delta i\left(x,y,t\right)\delta i\left(x,y,t+\tau \right)〉}{{〈i\left(x,y,t\right)〉}_{t}{〈i\left(x,y,t+\tau \right)〉}_{t+\tau }}\text{,}$$

where angular brackets denote spatial and temporal averaging and τ is the timelag between subsequent images. TICS analysis was performed using simFCS analysis software developed by the Laboratory for Fluorescence Dynamics, University of California, Irvine.

### Number & Brightness (N&B) analysis

The distribution and degree of aggregation of BChol in CHO cells was studied by number and brightness analysis (N&B) using ImageJ or simFCS [7,19]. The apparent brightness, *B*, is given as the ratio of variance and average of the intensity fluctuations, according to [19]:

(7) $$B=\frac{\sigma^{2}}{〈k〉}=\frac{\sum_{i}{\left(k_{i}-〈k〉\right)}^{2}}{\sum_{i}k_{i}}=\varepsilon +1.$$

Here, *k*_i_ is the number of photon counts at a particular pixel position, while 〈*k*〉 and σ^2^ represent the first and second moment of the intensity distribution (i.e., the average intensity and variance), respectively. The apparent brightness is, thus, a measure of the intensity variance per pixel normalized to the average intensity. It is related to the molecular brightness, ε, of the particles and is independent of the number of particles. The apparent number of particles, *N*, is calculated from the same parameters according to:

(8) $$N=\frac{{〈k〉}^{2}}{\sigma^{2}}=\frac{\varepsilon \cdot n}{\varepsilon +1}\text{.}$$

*N* is directly proportional to the number of fluorescent particles, n, in a given pixel location.

### Raster image correlation spectroscopy

Raster image correlation spectroscopy (RICS) was first described by Digman *et al*. as a method to measure the dynamics of particles moving fast in solution or in cells [16,17,61]. In raster scanning mode, the RICS autocorrelation function is given by:

(9) $$G_{S}\left(\xi ,\eta \right)=S\left(\xi ,\eta \right)\cdot G\left(\xi ,\eta \right)\text{,}$$

where *S*(*ξη*) is the correlation function due to the scanning of the laser beam and *G*(*ξη*) is the correlation function due to diffusion. In the temporal domain, the mean end-to-end distance of a random walk performed by a particle which is much smaller than the point spread function (PSF) will be proportional to the square root of time. Meanwhile, in the spatial domain the position of the particle as a function of time is described by a Gaussian distribution with a variance related to the diffusion coefficient. This means that on a LSM operating in the two-photon mode, where the laser is raster scanned across the sample line-by-line the temporal correlation function, *S*(*ξη*), has the following form:

(10) $$\text{S}\left(\xi ,\eta \right)=\text{exp}\left[-\frac{\frac{\delta r^{2}}{\omega_{0}^{2}}\left({\left|\xi \right|}^{2}+{\left|\eta \right|}^{2}\right)}{1+\frac{8D\left(\tau_{p}\left|\xi \right|+\tau_{l}\left|\eta \right|\right)}{\omega_{0}^{2}}}\right]\text{,}$$

where τ_p_ is the pixel residence time, τ_l_ is the line time and δ*r* is the pixel size. The spatial correlation function, *G*(*ξη*), is given by:

(11) $$G\left(\xi ,\eta \right)=\frac{\gamma }{N}{\left(1+\frac{8D\left(\tau_{p}\left|\xi \right|+\tau_{l}\left|\eta \right|\right)}{\omega_{0}^{2}}\right)}^{-3/2}\text{,}$$

where γ is a factor describing the geometry of the laser beam. In raster scanning mode, the correlation of the succeeding images is related on three different time scales. Along the horizontal direction the pixels are separated by the pixel dwell time (microseconds) while along the vertical direction the images are correlated by the line time (i.e., the time it takes to record the intensity of every pixel in a line plus the time it takes for the microscope to move to the next line). The line time is typically in milliseconds. Finally, the images are correlated by the time between two succeeding frames (seconds). Thus, RICS may be employed to measure the dynamics of particles with a wide range of diffusion coefficients. Diffusion maps and diffusion coefficients determined by RICS were calculated using simFCS software developed by the Laboratory for Fluorescence Dynamics, University of California, Irvine. [16,17].

## Competing interests

The authors declare that they have no competing interests.

## Authors’ contributions

FWL performed most image analysis and all supporting simulations with assistance of DW. The model for vesicle diffusivity (Eq. 1) was developed in collaboration between MAL, FWL and DW. LMS acquired two-photon microscopy image stacks for vesicle tracking. FWL wrote the main draft with substantial suggestions and comments from DW and MAL. RB synthesized BODIPY-cholesterol and commented on a late iteration of the manuscript. DW corrected the drafts and the final manuscript. Furthermore, DW performed the N&B analysis and acquired the images for RICS and TICS analysis. All authors read and approved the final manuscript.

## Supplementary Material

Additional file 1 **Figure S1.** Chemical structure of BODIPY-cholesterol (BChol) and dehydroergosterol (DHE). A, structure of BODIPY-cholesterol with the fluorophore (green, with light green underlay to highlight the fluorescent group) at carbon 24 of the sterol side chain. B, structure of DHE. Differences to cholesterol are indicated in green and red; i.e., an extra methyl group and double bond in the alkyl side chain and two additional double bonds in the steroid ring system. Only one additional double bond in the second ring distinguishes DHE from ergosterol (indicated in red). The three conjugated double bonds in the steroid ring (indicated in light blue) give DHE its slight fluorescence. **Figure S2**: Number and brightness analysis in HeLa cells labeled with BChol. Cells were repeatedly imaged on a 2P microscope with a pixel dwell time of 10 μsec and 100 frames in total. The apparent number (N) and brightness (B) of the BChol molecules were calculated from the intensity fluctuations per pixel positions, as described in the main text. A, the first 5 frames of the acquired sequence; B, N-map; C, B-map; D, intensity variance, (i.e., the second moment (2nd mt.) of the photon count distribution, as it enters in Eq. 6 in the main text. In addition, we calculated higher moments of the photon counting histogram using a plugin to Image J, kindly provided by Dr. Jay Unruh (Stowers Inst. for Microscopy, Kansas, USA). The 3rd, 4th and 5th moment were scaled identically using a FIRE LUT in the range of 0-950, and showed identical features. See text for further explanations. **Figure S3**: Photobleaching of BChol in CHO cells imaged with a wide field (WF) microscope (A) or a two-photon imaging system (B). In both cases 600 frames were acquired. Arrowheads in A indicate the BChol-labeled plasma membrane. The large central region in A is mostly slowly bleaching autofluorescence. C, quantification of BChol’s intensity for the WF sequence (light grey line, data and darkgrey line, monoexp. fit) and of the 2P sequence (black line). D, quantification of BChol’s intensity for another WF sequence with higher illumination intensity (grey symbols, data and grey line, fit); and the 2P sequence of Fig. 2 in the main text. **Figure S4**: Result of the reaction-diffusion simulation with diffusing particles shown in blue and binders shown in red. **Figure S5**: Analysis of the simulated data. Panel A shows the first frame of the simulated image stack. Panels B, C and D shows the diffusion map, the number of particles per pixel and the brightness of each pixel, respectively. By comparison with Figure 2 (in the main text) we can see that the simulation of Brownian diffusion with transient binding faithfully resembles the experimental data. The diffusion constant is lower in the areas with binding macromolecular structures, the majority of diffusing molecules are found in the area with binders and the brightness map shows a low degree of aggregation. **Figure S6**: Distribution of diffusing particles with and without exchange between the binders and the free particles with and without binding, A and D, respectively. B and E are maps of the apparent number of molecules (N), and C and F show maps of the apparent brightness (B). **Figure S7**: Simulation with increased mobility of the free particles. The binding and dissociation rate constants were kept at kon = 0.23 s^-1^ and koff = 0.5 s^-1^, while the diffusion coefficient of the particles (simulating sterol monomers was increased from 1 μm^2^/s to 300 μm^2^/s). Distribution of diffusing particles is shown in A, while B and C are maps of the apparent number of molecules (N), and the apparent brightness (B), respectively. **Figure S8**: Simulation with stronger binding: All settings were as described in Fig. S5 except for the unbinding rate constant, koff, which was reduced from 0.5 s^-1^ to 0.05 s^-1^. Here 5573 particles were bound to 100 binders compared to 278 particles in the previous experiment. Therefore, the bound particles (or vesicles) become more visible Distribution of diffusing particles is in A, while B and C are maps of the apparent number of molecules (N), and the apparent brightness (B), respectively. **Figure S9**: 2P time-lapse sequence showing shuttling of a small vesicle (in green) from a donor to an acceptor vesicle (from bottom to top of the image frames). The lower donor vesicle becomes first elongated, followed by formation and fission of a small vesicle which shuttles to and fuses with a stationary acceptor vesicle. Images in this example sequence were acquired every 4 s. The size of the frames is 1.73 x 2.59 μm. **Figure S10**: From the end-to-end distance of a trajectory one may estimate the degree of directed motion. That is, if a vesicle is subject to directed motion it is expected to traverse further away from the starting point than a vesicle which is not subject to directed motion. Panels A-C show the distribution of end-to-end distances in control cells (A) and in cells with either disrupted microtubules (B) or actin filaments (C). Also, shown in panel D is the mean end-to-end distance at each condition which further emphasize the decreased vesicle mobility upon disruption of the cytoskeletal filaments. Click here for file
