# Supplementary material for: Two-photon time-lapse microscopy of BODIPY-cholesterol reveals anomalous sterol diffusion in chinese hamster ovary cells
Source: BMC Biophys. 2012 Oct 18;5:20. doi: 10.1186/2046-1682-5-20 (PMC3532368; doi:10.1186/2046-1682-5-20)
Supplement: Additional file 1 — Figure S1. Chemical structure of BODIPY-cholesterol (BChol) and dehydroergosterol (DHE). A, structure of BODIPY-cholesterol with the fluorophore (green, with light green underlay to highlight the fluorescent group) at carbon 24 of the sterol side chain. B, structure of DHE. Differences to cholesterol are indicated in green and red; i.e., an extra methyl group and double bond in the alkyl side chain and two additional double bonds in the steroid ring system. Only one additional double bond in the second ring distinguishes DHE from ergosterol (indicated in red). The three conjugated double bonds in the steroid ring (indicated in light blue) give DHE its slight fluorescence. Figure S2: Number and brightness analysis in HeLa cells labeled with BChol. Cells were repeatedly imaged on a 2P microscope with a pixel dwell time of 10 μsec and 100 frames in total. The apparent number (N) and brightness (B) of the BChol molecules were calculated from the intensity fluctuations per pixel positions, as described in the main text. A, the first 5 frames of the acquired sequence; B, N-map; C, B-map; D, intensity variance, (i.e., the second moment (2nd mt.) of the photon count distribution, as it enters in Eq. 6 in the main text. In addition, we calculated higher moments of the photon counting histogram using a plugin to Image J, kindly provided by Dr. Jay Unruh (Stowers Inst. for Microscopy, Kansas, USA). The 3rd, 4th and 5th moment were scaled identically using a FIRE LUT in the range of 0-950, and showed identical features. See text for further explanations. Figure S3: Photobleaching of BChol in CHO cells imaged with a wide field (WF) microscope (A) or a two-photon imaging system (B). In both cases 600 frames were acquired. Arrowheads in A indicate the BChol-labeled plasma membrane. The large central region in A is mostly slowly bleaching autofluorescence. C, quantification of BChol’s intensity for the WF sequence (light grey line, data and darkgrey line, monoexp. fit) and of the 2 [file 2046-1682-5-20-S1.pdf]

Supplementary Material for ‘Two-Photon Time-Lapse Microscopy of BODIPY-Cholesterol Reveals Anomalous Sterol Diffusion in Chinese Hamster Ovary Cells’

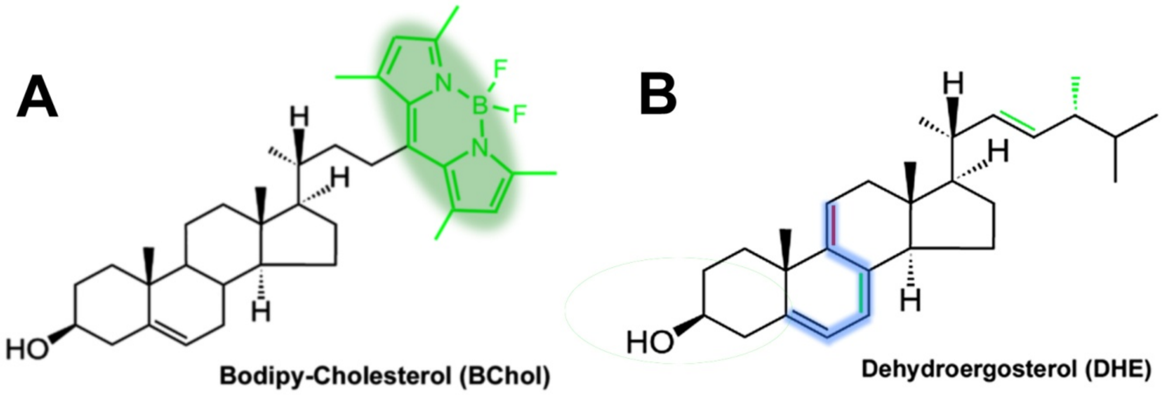

Figure S 1: Chemical structure of BODIPY-cholesterol (BChol) and dehydroergosterol (DHE). A, structure of BODIPY-cholesterol with the fluorophore (green, with light green underlay to highlight the fluorescent group) at carbon 24 of the sterol side chain. B, structure of DHE. Differences to cholesterol are indicated in green and red; i.e., an extra methyl group and double bond in the alkyl side chain and two additional double bonds in the steroid ring system. Only one additional double bond in the second ring distinguishes DHE from ergosterol (indicated in red). The three conjugated double bonds in the steroid ring (indicated in light blue) give DHE its slight fluorescence.

Number & Brightness analysis of BChol by two-photon microscopy in HeLa cells

To rule out any cell-type specific effects, we performed a N&B analysis additionally in HeLa cells labeled with BChol, as described in the main text for CHO cells. Here, we also found a homogeneous B-map suggesting that all BChol in the plasma membrane exists as monomers (Fig. S2C). A histogram of B-values revealed a single population with a mean value slightly larger than one (not shown). This is in full accordance with one mobile population of BChol molecules in the membrane (see Ref. 20 in the main text). If the brightness is exactly one, the only contribution to the photon counting distribution will be the photon shot noise, since that is Poisson distributed with mean value equal to variance. That scenario is characteristic for completely immobile fluorescent molecules, but that was not found here. If molecular diffusion contributes to the intensity fluctuations and the only diffusing species is in a monomer form, the photon counting histogram is broadened and the variance gets a little larger than the mean, giving a B-map with values of slightly > 1. This is, what we observed for BChol in both, CHO cells (Fig. 2D) and HeLa cells (Fig. S2C). If the diffusing species can additionally form aggregates, the variance of the fluctuating signal will be

further increased resulting in a second (or higher) population and visible features in the B-map. This was not observed for BChol in both cell types.

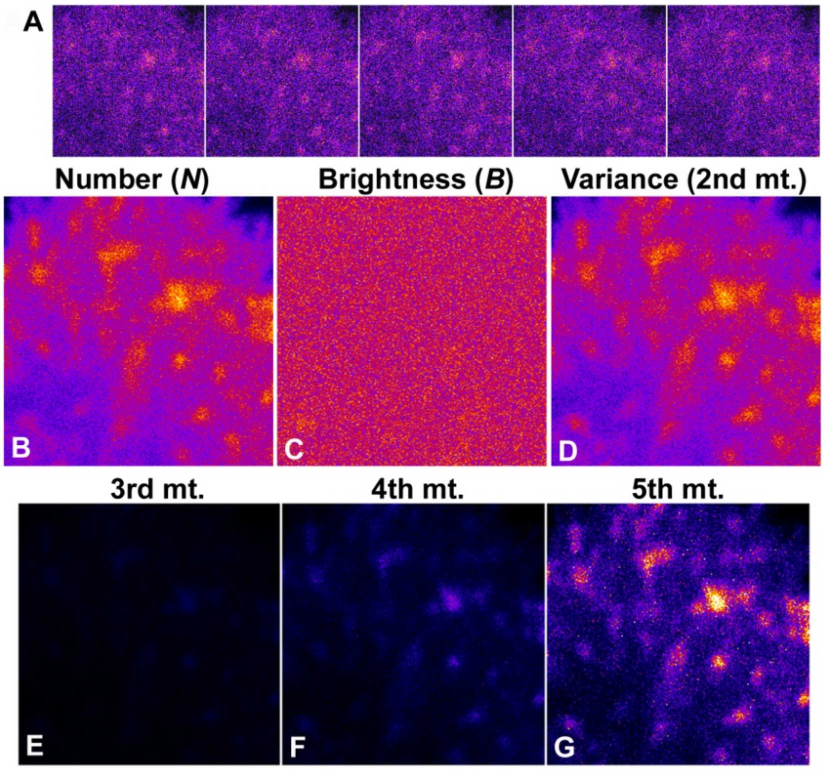

Figure S 2: Number and brightness analysis in HeLa cells labeled with BChol. Cells were repeatedly imaged on a 2P microscope with a pixel dwell time of 10 μsec and 100 frames in total. The apparent number (N) and brightness (B) of the BChol molecules were calculated from the intensity fluctuations per pixel positions, as described in the main text. A, the first 5 frames of the acquired sequence; B, N-map; C, B-map; D, intensity variance, (i.e., the second moment (2nd mt.) of the photon count distribution, as it enters in Eq. 6 in the main text. In addition, we calculated higher moments of the photon counting histogram using a plugin to Image J, kindly provided by Dr. Jay Unruh (Stowers Inst. for Microscopy, Kansas, USA). The 3rd, 4th and 5th moment were scaled identically using a FIRE LUT in the range of 0-950, and showed identical features. See text for further explanations.

The N&B analysis is conceptually based on the calculation of statistical moments of the fluorescence intensity fluctuations in the focal volume of a laser scanning based imaging system (see Ref. 20 and 21 in the main text and Qian and Elson, 1990). The central moments of a statistical distribution (i.e. moments around the mean value) provide the variance (second moment), the skewness (3rd moment) etc. and give a complete description of the distribution. Qian and Elson (1990) have shown that further information about eventual fluorophore aggregates including their stoichiometry can be inferred from higher moments of the fluctuating fluorescence signal (Qian and Elson, 1990). Thus, eventual aggregates of BChol might become visible in maps of the higher moments of the measured fluorescence series. We calculated moments up to the 5th order for the image sequence of BChol-labeled HeLa cells, and found no change in the features (Fig. S2E-G). Together, the results of this analysis confirm that BChol exists as mobile monomers in the plasma membrane of living mammalian cells.

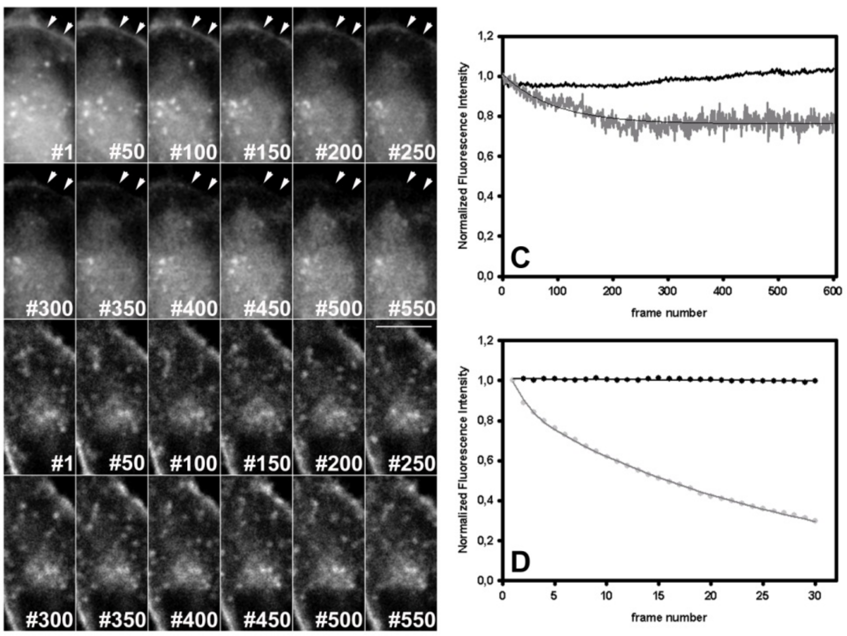

Figure S 3: Photobleaching of BChol in CHO cells imaged with a wide field (WF) microscope (A) or a two-photon imaging system (B). In both cases 600 frames were acquired. Arrowheads in A indicate the BChol-labeled plasma membrane. The large central region in A is mostly slowly bleaching autofluorescence. C, quantification of BChol's intensity for the WF sequence (light grey line, data and dark grey line, monoexp. fit) and of the 2P sequence (black line). D, quantification of BChol's intensity for another WF sequence with higher illumination intensity (grey symbols, data and grey line, fit); and the 2P sequence of Fig. 2 in the main text.

Comparative analysis of photobleaching of BChol in wide field and two-photon microscopy

CHO cells were labeled with BChol, as described in the main text and imaged either on a wide field (WF) microscope equipped with an Andor EMCCD (A, C) or equipped with a Hamamatsu back-thinned CCD camera, Orca 2.0 (D) or on a two-photon (2P) microscope (B-D). For the experiments shown in Fig. S3A-C, a three-fold lower labeling amount than usual was used. The WF sequence of Fig. S3A was acquired using a neutral density filter of 50%, and the 2P sequence with a power of 0.5 mW of the MaiTai laser tuned to 930 nm. BChol bleaches rapidly in the WF modus, as indicated by the decaying intensity in the plasma membrane (arrows in Fig. S3A) and in some perinuclear vesicles. In that example, there was a rather high fraction of autfluorescence, which did not bleach significantly during the image acquisition. Quantification of cell-associated fluorescence in the green emission channel (BChol and autofluorescence) is shown in Fig. S3C (light grey line) and this data was fitted with a monoexponential decay function including residuum of the form:

$$\mathrm{f}(n) = A\exp(-kn) + \mathrm{f}_0,$$

where fo is the non-bleaching fraction, n, the number of frames, k the bleach rate constant and A is the amplitude of the bleaching BChol fluorescence (dark grey line). From the estimated bleach rate constant, we could infer a bleach 'half-time' $t_{1/2}$=ln2/k=64.2 frames. In contrast, a corresponding 2P

image sequence also 600 frames long, did not show any photobleaching of BChol (Fig. S3B). Quantification of cell-associated fluorescence in the green emission channel showed even a slight increase in fluorescence which stems from an increased number of sterol vesicles accumulating in the quantified cell region over time (Fig. S3C, black line). Another example is shown in Fig. S3D, where in total 70 frames were acquired either on a WF microscope without neutral density filter and 200 msec acquisition time (grey symbols, data; dark grey line, biexp. fit without residuum of the form:

$$f(n) = A_1 \exp(-k_1 n) + A_2 \exp(-k_2 n),$$

or on a 2P microscope, that time with 1.5 mW laser power (black symbols, data; black line, monoexp. fit without residuum of the form:

$$f(n) = A \exp(-kn).$$

Due to the more intense illumination, BChol bleached even stronger in the WF sequence giving an amplitude-weighted half time of $t_{1/2}$=18.8 frames. In the corresponding two-photon sequence, there was an insignificant photobleaching of BChol giving a bleach ´half-time’ of $t_{1/2}$=1900 frames. In summary, photobleaching of BChol can be safely ignored in our two-photon set up. Photobleaching of BChol in conventional confocal microscopy was compare to that in WF imaging (not shown).

Simulation of Brownian Diffusion with Transient Binding

Intracellular binding of proteins to macromolecular structures leads to a heterogeneous distribution of the fluophore as well as to a lower “effective” diffusion constant. Since BChol is highly hydrophobic, it is unlikely that it is found free in the cytoplasm. Thus, the non-vesicular transport of BChol most likely occurs via one or more sterol transport proteins. Furthermore, the cytoplasm is crowded with vesicles as well as organelles which all potentially could bind BChol transiently. Using a stochastic simulation of diffusion with binding we found a fluophore distribution similar to the one observed for BChol. This further strengthens that the aggregation and lower diffusion constant in the crowded cytoplasm is caused by transient binding events. The simulated model is a stochastic reaction-diffusion model where a diffusing particle may change between a free state and a bound state according to a rate constant for binding, k_{on}, and a rate constant for release, k_{off}. Thus, assuming that a freely diffusing particle is within the binding radius of a binder, the probability of binding is given by $P_B = k_{on}/(k_{on} + k_{off})$. Similarly, the probability for a bound particle to escape the

binder is given by $P_F = k_{off}/(k_{on} + k_{off})$. Finally, the model includes an option to set the time between frames using the parameter *dt*.

To compare the model with the experimental results the simulation was performed with a random distribution of 20,000 molecules moving by Brownian diffusion with a diffusion constant of 1 $\mu m^2/s$. In the lower left quadrant of the simulated area 100 binders with a diffusion constant of 0.1 $\mu m^2/s$ were placed randomly. The binders had a diameter of 450 nm, corresponding to the average size of BChol containing vesicles (see main text figure 4). The reaction rates were set to $k_{on} = 0.23$ s^{-1} and $k_{off} = 0.5$ s^{-1}. The pixel size was set to 0.1 µm/pixel while the sampling time was set to 10 frames per second. Figure S4 shows the output of the simulation with diffusing particles and binders shown in blue and red, respectively.

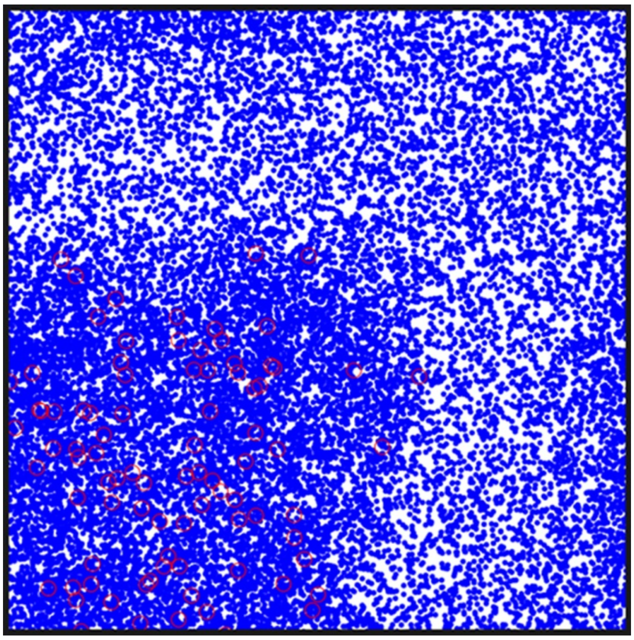

Figure S 4: Result of the reaction-diffusion simulation with diffusing particles shown in blue and binders shown in red.

During the simulation, images were digitalized in a 256x256 pixel format, convolved with a Gauss filter to mimic the blurring by the microscope optics and saved for further analysis as shown in Fig. S5A. Notice that in the generated images the binders are not visible. Subsequently, the image stack was analyzed by temporal image correlation spectroscopy (TICS) to determine a map of diffusion constants (Fig. S2B). In addition, a number and brightness (*N&B*) analysis was performed to calculate the number of molecules per pixel (*N*-map) and the brightness per pixel (*B*-map) (Fig. S5C and D). Note that in these calculations the only contribution to a ‘fluctuating signal’ is the movement of the particles. This is a simple stochastic process. Real flurophores in cells will

additionally emit photons when they relax from the excited state, which together with the motion of the molecules gives a double stochastic process (see for example, Ref. 20 in the main text). Since we assumed that the Poisson distribution of photon emission is identical for all simulated molecules (i.e., the fluorophores do not interact (i.e., no self-quenching etc.), the photon shot noise can be neglected (see the same strategy in other studies, for example in Qian and Elson, 1990).

The simulated image as well as the *N*-map shows that the diffusing particles are mainly located in the lower left quadrant where the binders are found. At the same time the *B*-map is relatively homogeneous showing that the particles do not aggregate. Finally, the map of diffusion constants show that diffusion is significantly slower in the area containing binders compared to the area with no binders. Comparing these results to the experimental results presented in figure 2 (in the main text), the similarity is evident. Thus, the simulation suggests that the spatially heterogeneous diffusion constants of BChol are a consequence of binding of BChol to immobile or slowly diffusing intracellular binders.

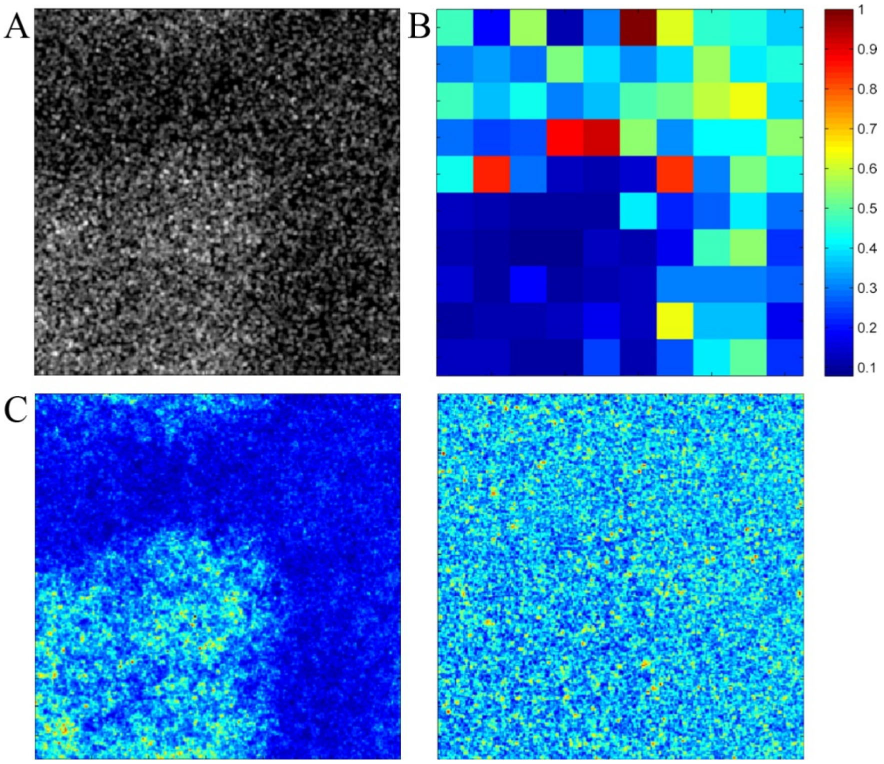

Figure S 5: Analysis of the simulated data. Panel A shows the first frame of the simulated image stack. Panels B, C and D shows the diffusion map, the number of particles per pixel and the brightness of each pixel, respectively. By comparison with Figure 2 (in the main text) we can see that the simulation of Brownian diffusion with transient binding faithfully resembles the experimental data. The diffusion constant is lower in the areas with binding macromolecular structures, the majority of diffusing molecules are found in the area with binders and the brightness map shows a low degree of aggregation.

Alternatively, it might be possible that the experimental data on *N&B* analysis and diffusion maps of BChol are caused by sterol containing vesicles and sterol monomers not interacting with each other. To test this hypothesis, we ran a second simulation of a population of free particles (non-vesicular) and another population of bound (vesicular) particles without any interaction between both populations. This second simulation was run using the same program but with input from the first simulation (i.e., information about particle and binder positions and which particles are bound to which binder in the last iteration) but all binding/dissociation rate constants set to zero (see Fig S6 for a comparison between the two simulations). Both simulations had a total of 278 bound particles.

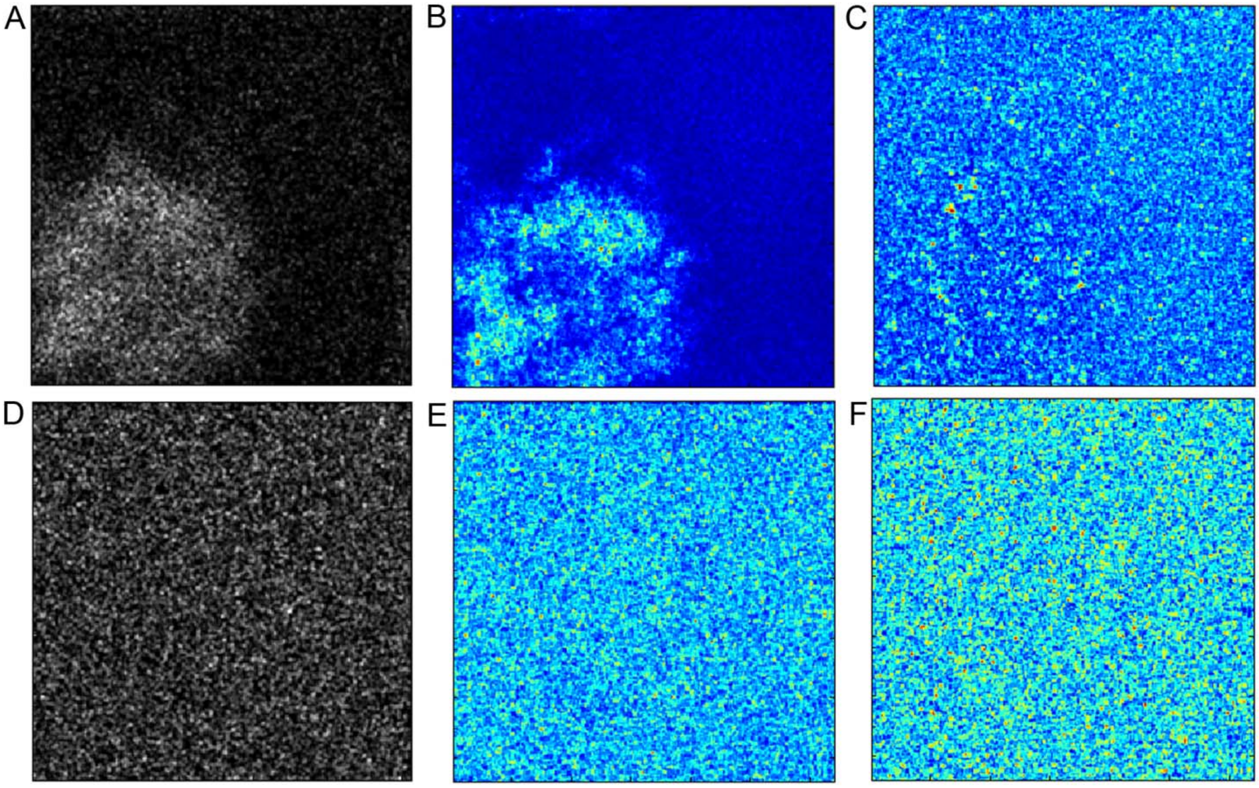

Figure S 6: Distribution of diffusing particles with and without exchange between the binders and the free particles with and without binding, A and D, respectively. B and E are maps of the apparent number of molecules (*N*), and C and F show maps of the apparent brightness (*B*).

Interestingly, this computer experiment shows that the distribution of particles in the simulated areas is very different in the presence or absence of reversible binding: while the particles cluster near the binders in the lower left quadrant when exchange is possible (Fig. S6A and S5), they are evenly distributed over the area when there is no exchange (Fig. S6B). This suggests that transient binding of the particles to binders has an indirect effect on the mobility of the particles, which can be illustrated as follows. When transiently bound particles become released from the less mobile

binders, there is a high probability that the particles will rebind before they can escape the binders reach by diffusion. As a consequence, even free particles will tend to cluster near the slowly moving binders. On the other hand, when there is no interaction between the particles and the binders, the free particles will distribute evenly over the area until eventual concentration gradients vanish, in accordance with diffusion theory. To test this idea further, we performed another simulation where the diffusion constant of the free particles was increased from 1 μm^2/s to 300 μm^2/s, while the binding and release parameters were kept constant at the values of the first simulation (i.e. k_{on} = 0.23 s^{-1} and k_{off} = 0.5 s^{-1}). Comparing Fig. S 7 with Fig. S 6A shows that when the free particles are more mobile, significantly fewer particles are found in the lower left quadrant where the binders are found. Thus, an increase in mobility raises the probability for a particle to escape the binding region of the much slower moving binders.

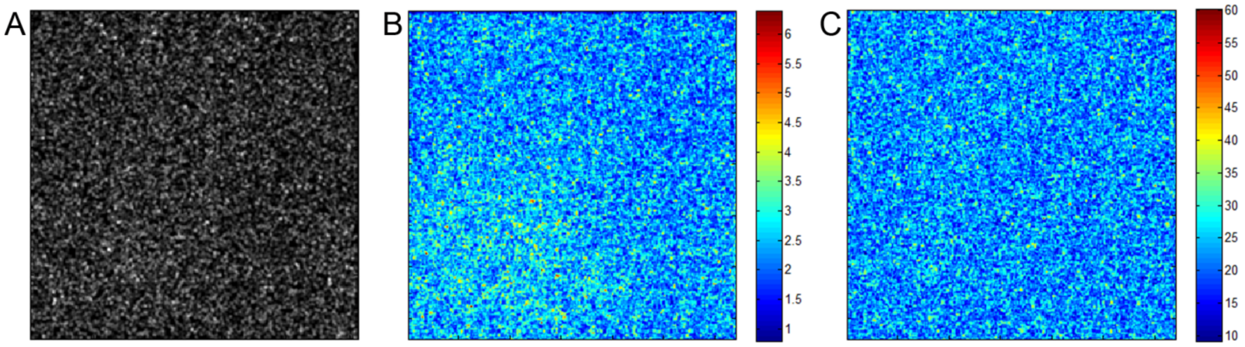

Figure S 7: Simulation with increased mobility of the free particles. The binding and dissociation rate constants were kept at k_{on} = 0.23 s^{-1} and k_{off} = 0.5 s^{-1}, while the diffusion coefficient of the particles (simulating sterol monomers was increased from 1 μm^2/s to 300 μm^2/s). Distribution of diffusing particles is shown in A, while B and C are maps of the apparent number of molecules (*N*), and the apparent brightness (*B*), respectively.

Finally, we simulated the opposite scenario, where binding strength of the particles to the binders was increased 10-fold, while the diffusion constant of the particles was kept low at 1 μm^2/s. As expected, this resulted in a clustered distribution of particles resembling that of the binders, and a very structured B-map (see Fig. S8, below).

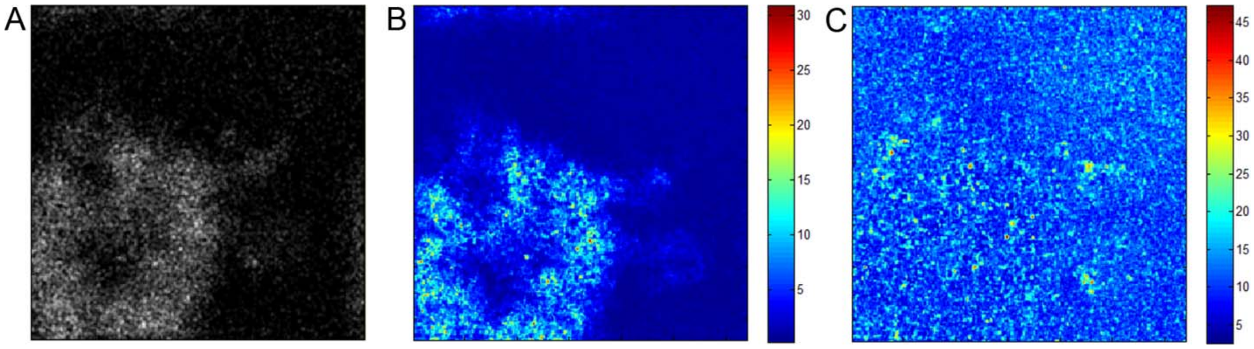

Fig. S8: Simulation with stronger binding: All settings were as described in Fig. S5 except for the unbinding rate constant, k_{off}, which was reduced from 0.5 s^{-1} to 0.05 s^{-1}. Here 5573 particles were bound to 100 binders compared to 278 particles in the previous experiment. Therefore, the bound particles (or vesicles) become more visible Distribution of diffusing particles is in A, while B and C are maps of the apparent number of molecules (*N*), and the apparent brightness (*B*), respectively.

Together, the simulations suggest that transient binding of BChol to immobile or slowly diffusing intracellular membranes results in the observed heterogeneous sterol diffusion in living cells.

Observation of shuttling sterol vesicles between stationary vesicles containing BChol

Occasionally, we observed small vesicles containing BChol with a diameter of approx. 0.32 μm moving between larger, rather immobile sterol vesicles having a diameter of ~0.5-0.65 μm (Fig. S9, below). The peak intensity of BChol fluorescence in the small shuttling 'carrier' vesicles was about one third to one half of that in the immobile donor- and acceptor vesicles, respectively. Since the shuttling vesicles were smaller, the estimated integrated intensity of BChol was about one fifth in these vesicles compared to the donor- and acceptor vesicles.

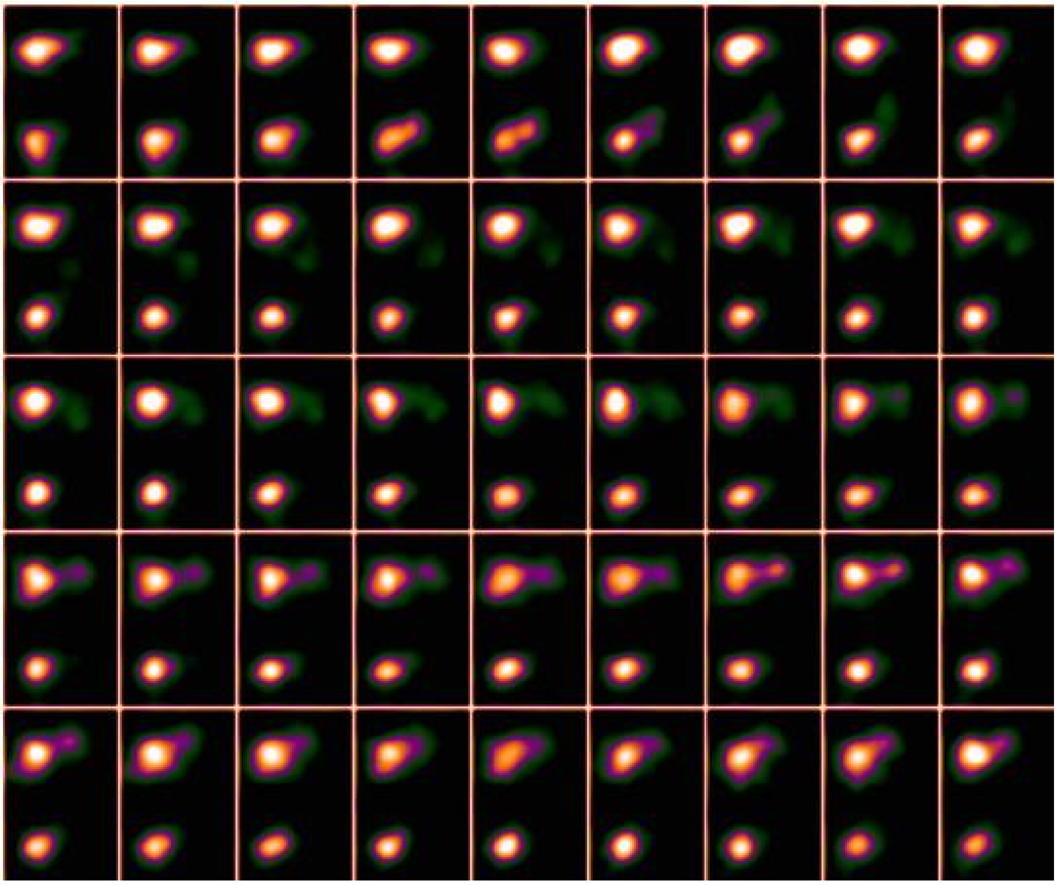

Figure S 9: 2P time-lapse sequence showing shuttling of a small vesicle (in green) from a donor to an acceptor vesicle (from bottom to top of the image frames). The lower donor vesicle becomes first elongated, followed by formation and fission of a small vesicle which shuttles to and fuses with a stationary acceptor vesicle. Images in this example sequence were acquired every 4 sec. The size of the frames is 1.73 x 2.59 μm

End-to-end distances of vesicle trajectories as a measure of directed motion

The end-to-end distance of a vesicle trajectory may be used as a rough measure of whether the vesicle is subject to directed motion or Brownian motion/restricted motion. For examples, see the trajectories in Fig. 4E (in the main text). Notice, however, that the end-to-end distance as a measure of directed transport is only valid if the active transport occur in one direction. If, for example, the vesicle moves to one location and subsequently returns to a position close to the starting point, the end-to-end distance will be small, although the vesicle is subject to ballistic motion. To ensure that this was not the case, we verified by visual inspection that none of the vesicles moved in this way.

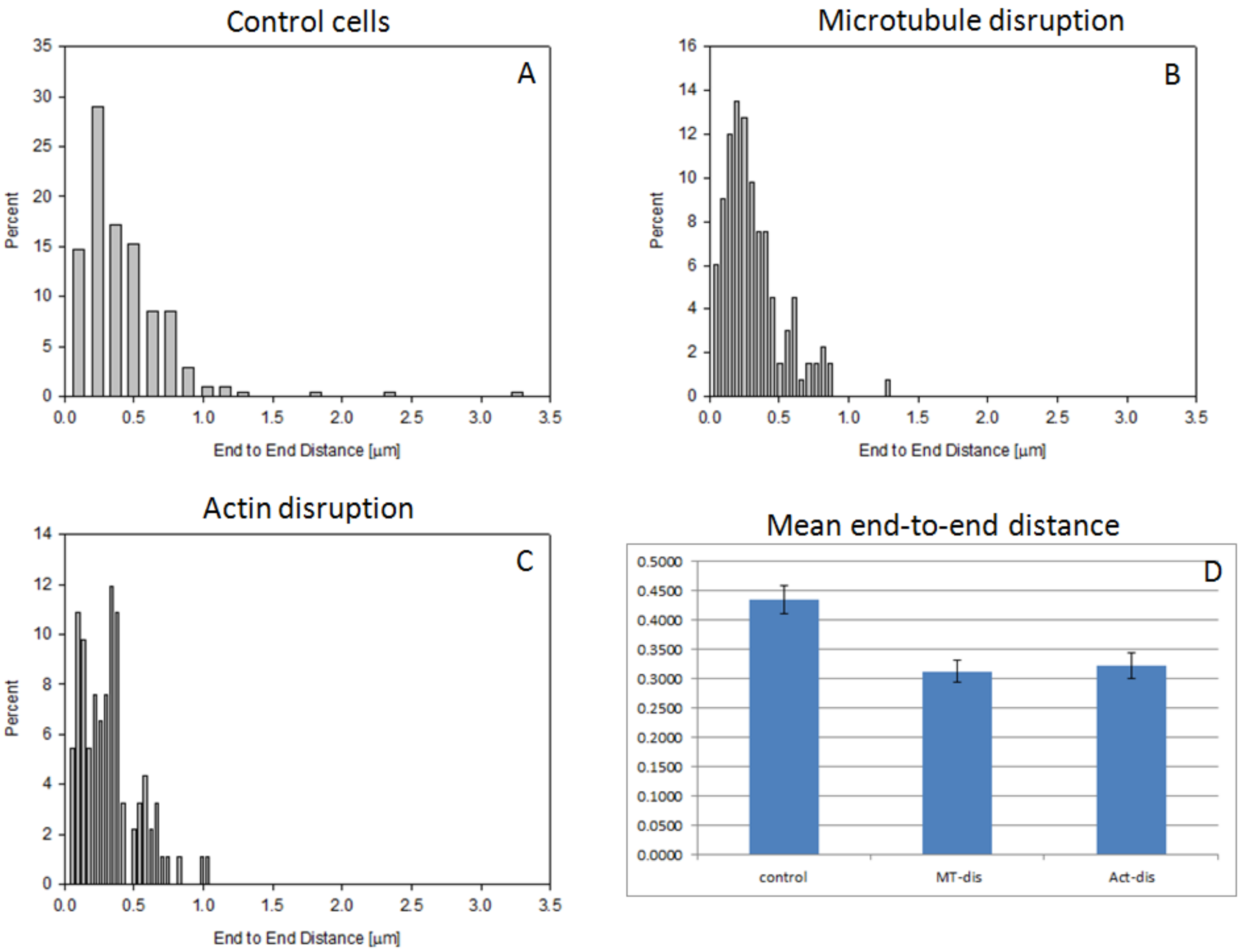

Figure S 10: From the end-to-end distance of a trajectory one may estimate the degree of directed motion. That is, if a vesicle is subject to directed motion it is expected to traverse further away from the starting point than a vesicle which is not subject to directed motion. Panels A-C show the distribution of end-to-end distances in control cells (A) and in cells with either disrupted microtubules (B) or actin filaments (C). Also, shown in panel D is the mean end-to-end distance at each condition which further emphasize the decreased vesicle mobility upon disruption of the cytoskeletal filaments.

To determine, whether the transition from anomalous subdiffusion to directed motion is caused by a small but statistically significant group of vesicles or, alternatively the majority of the vesicles

indeed move with a very low velocity, we calculated the end-to-end distance of each trajectory. This is shown in Fig. S10A-C for control cells (A), and in cells with disrupted microtubules (B) or actin filaments (C). From this we can see that the majority of vesicles have an end-to-end distance smaller than 1 μm. Out of 210 vesicles tracked in control cells only eight had an end-to-end distance larger than 1 μm. Furthermore, we found that upon disruption of the cytoskeletal filaments the end-to-end distances become smaller which is further reflected in the mean end-to-end distance shown in Fig. S10D. This is consistent with the observation that after disruption of microtubules or actin filaments the vesicles become less mobile and the velocity of active transport decreases.

Additional reference

Qian, H. and Elson, E.L., 1990. **On the analysis of high order moments of fluorescence fluctuations.** Biophys. J. **57**:375-380.

Matlab Code for the Simulation of Diffusion with Transient Binding

The matlab code for the simulation is presented below with comments. Please notice that the program calls the matlab function hist2.m by Lazio Balkay which can be downloaded from the MATLAB File Exchange.

```
function[x,y,histmat,Bmat,im] = diffBind(Npar,Nbind,s,D,Dbinder,kon,koff,pix_size,dt,r,it)
%Simulates diffusion of particles with transient binding to mobile or stationary binders in a 2D square area with periodic boundaries. The pixel size (i.e., um/pixel) and %time between frames are adjustable. To get a reliable output the time per frame should not be too large with respect to the fastest diffusion constant.
%
%Input variables
%Npar number of diffusing particles
%Nbind number of binders
%s size of square is s x s
%D Diffusion constant of particles
%Dbinder Diffusion constant of binders
%kon on-rate for binding
%koff off rate for binding
%pix_size um/pixel
%dt time between frames
%r binding radius
%it number of frames
%
%Output variables
%x x-coordinates for particles as a function of time (one row per time point)
%y y-coordinates for particles as a function of time (one row per time point)

%Reserve space in memory for x- and y-matrices
x = zeros(Npar,it);
y = zeros(Npar,it);
```

```
%Distribute diffusing particles randomly in square
x_ini = rand(Npar,1)*s;
y_ini = rand(Npar,1)*s;
x(:,1) = x_ini;
y(:,1) = y_ini;

%Distribute binders randomnly within square
x_bind = zeros(Nbind,it);
y_bind = zeros(Nbind,it);

x_b = rand(Nbind,1)*s/2;
y_b = rand(Nbind,1)*s/2;

x_bind(:,1) = x_b;
y_bind(:,1) = y_b;

%Generate random steps for diffusing particles
Rx = randn(Npar,it).*sqrt(2*D*dt).*(1/pix_size);
Ry = randn(Npar,it).*sqrt(2*D*dt).*(1/pix_size);

%Generate random steps for diffusing binders
Rxbind = randn(Nbind,it).*sqrt(2*Dbinder*dt).*(1/pix_size);
Rybind = randn(Nbind,it).*sqrt(2*Dbinder*dt).*(1/pix_size);

%Correct radius of binding for micrometer/pixel
r = r*(1/pix_size);

%Generate matrix showing which particles are bound and to which binder. The
%matrix contains Npar rows and Nbind columns. If e.g. particle 10 binds to
%binder 5 Bmat(10,5) is set to one. Then if the particle later unbinds
%Bmat(10,5) is reset to zero.
Bmat = zeros(Npar,Nbind);

%Perform diffusion
for i = 2:it
   x(:,i) = x(:,i-1)+Rx(:,i);
   y(:,i) = y(:,i-1)+Ry(:,i);

   x_bind(:,i) = x_bind(:,i-1)+Rxbind(:,i);
   y_bind(:,i) = y_bind(:,i-1)+Rybind(:,i);


%Find potentially binding particles
for j = 1:Nbind
r_bind1 = sqrt((x(:,i-1)-x_bind(j,i-1)).^2+(y(:,i-1)-y_bind(j,i-1)).^2);
       r_bind2 = sqrt((x(:,i)-x_bind(j,i)).^2+(y(:,i)-y_bind(j,i)).^2);
B = find(r_bind1 > r);
for m = 1:numel(B)
if r_bind2(B(m)) <= r && rand <= kon/(kon+koff)
               Bmat(B(m),j) = 1;
               Rx(B(m),:) = 0;
               Ry(B(m),:) = 0;
```

```
end
end

%Find potentially unbinding particles
        B = find(Bmat(:,j) == 1);
for m = 1:numel(B)
if rand <= koff/(kon+koff)
%if r_bind2(B(m)) > r && rand <= koff/(kon+koff)
                Bmat(B(m),j) = 0;
                Rx(B(m),:) = randn(1,it).*sqrt(2*D*dt);
                Ry(B(m),:) = randn(1,it).*sqrt(2*D*dt);
end
end


end

%Enforce periodic boundaries
    F = find(x > s);
    x(F) = (x(F)-s);
    F = find(y > s);
    y(F) = (y(F)-s);

    F = find(x < 0);
    x(F) = s+(x(F));
    F = find(y < 0);
    y(F) = s+(y(F));

end

%For each position update an image is generated as a 2D histogram of the number of particles per pixel. Subsequently, the 2D histogram is displayed as an image and this image is grapped from the screen and saved in the matrix, im.
histmat = zeros(s,s,it);
figure
for i = 1:it
hism  = hist2(x(:,i), y(:,i), 0:1:s-1, 0:1:s-1);
histmat(:,:,i) = hism;
H = fspecial('gaussian',[3 3],1.0);
histm = imfilter(histmat,H);
imagesc(histm(:,:,i));axis image;colormap(gray)
set(gca,'YDir','normal')
frames(i) = getframe;
pause(0.001)
end

im_temp = frames(1).cdata;
[xi,yi,~] = size(im_temp);
im = zeros(xi,yi,it);

for i = 1:it
    im_temp = frames(i).cdata;
    im(:,:,i) = im_temp(:,:,1);
```

```
end

%Show plot of particles in blue and binders in red.
figure,plot(x(:,it),y(:,it),'b.','MarkerSize',7);
hold on
plot(x_bind(:,it),y_bind(:,it),'ro')
axis([0 s 0 s]);
axis square
hold off
```
